# Droplet-based high-throughput 3D genome structure mapping of single cells with simultaneous transcriptomics

**DOI:** 10.1038/s41421-025-00770-8

**Published:** 2025-01-21

**Authors:** Honggui Wu, Maoxu Wang, Yinghui Zheng, X. Sunney Xie

**Affiliations:** 1 Biomedical Pioneering Innovation Center (BIOPIC), and School of Life Sciences, Peking University, Beijing, China; 2 Changping Laboratory, Beijing, China; 3 Present Address: Broad Institute of MIT and Harvard, Cambridge, MA USA

**Keywords:** Chromatin analysis, Chromatin structure

## Abstract

Single-cell three-dimensional (3D) genome techniques have advanced our understanding of cell-type-specific chromatin structures in complex tissues, yet current methodologies are limited in cell throughput. Here we introduce a high-throughput single-cell Hi-C (dscHi-C) approach and its transcriptome co-assay (dscHi-C-multiome) using droplet microfluidics. Using dscHi-C, we investigate chromatin structural changes during mouse brain aging by profiling 32,777 single cells across three developmental stages (3 months, 12 months, and 23 months), yielding a median of 78,220 unique contacts. Our results show that genes with significant structural changes are enriched in pathways related to metabolic process and morphology change in neurons, and innate immune response in glial cells, highlighting the role of 3D genome organization in physiological brain aging. Furthermore, our multi-omics joint assay, dscHi-C-multiome, enables precise cell type identification in the adult mouse brain and uncovers the intricate relationship between genome architecture and gene expression. Collectively, we developed the sensitive, high-throughput dscHi-C and its multi-omics derivative, dscHi-C-multiome, demonstrating their potential for large-scale cell atlas studies in development and disease.

## Introduction

Chromatin structure is fundamental to the DNA-templated process, serving as a critical layer for gene regulation. Throughout development and disease progression, chromatin structure undergoes significant reorganization^1,2^ and exhibits cell type-specific configurations. Techniques such as chromosome conformation capture and its derivatives have advanced our understanding of chromatin folding^3–5^. Over the past decade, several single-cell three-dimensional (3D) genome mapping techniques have emerged^6–10^, enabling the identification of cell type-specific chromatin structural changes within tissues during both developmental and disease processes^1,11–13^. However, current scHi-C methodologies are constrained by limited cell throughput. Although high-throughput scHi-C methods using combinatorial indexing have been introduced^14,15^, these approaches are often labor-intensive, require custom reagents, and yield sparse data characterized by a low number of contacts.

Aging is a universal process marked by a progressive decline in physiological function that have broad impacts at cellular and systemic level^16,17^. Despite its broad effects, the underlying mechanisms driving aging remain poorly understood. Notably, aging contributes to the deterioration of brain function, leading to cognitive decline and an increased susceptibility to neurodegenerative diseases such as Alzheimer’s and Parkinson’s disease^18,19^. While single-cell genomics has revolutionized aging research by providing unprecedented insights into cellular and molecular changes^20–23^—such as the widespread activation of glial and immune cells and aging-induced inflammation—the alterations in chromatin architecture during aging have been less thoroughly characterized^11,24^. Recent scHi-C studies have highlighted lifelong chromatin structural reorganizations in granule cells of the cerebellum^11^; however, the dynamics of chromatin structure changes in the cerebrum during aging remain largely unexamined.

**Fig. 1:**
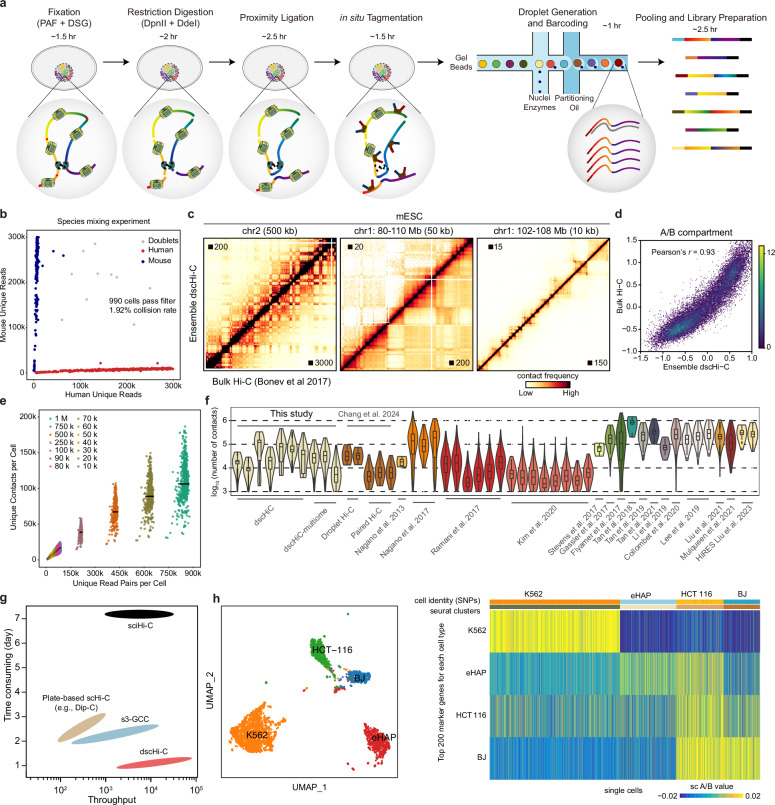
Droplet-based scHi-C assay. **a** Schematics of dscHi-C workflow. **b** Unique DNA reads mapping to the human and mouse genome. Species mixing experiment was performed using a mixture of human (GM12878) and mouse (mESC) cells to assess the collision rate. **c** Contact maps of ensemble dscHi-C and bulk Hi-C data for mESC at 500, 50, and 10-kb resolution, respectively. **d** Density plot showing the correlation of A/B compartment between ensemble dscHi-C and bulk Hi-C. **e** Scatter plot of downsample analysis showing the relationship between raw reads and unique contacts per cell of dscHi-C. The central line indicates the median value (*n* = 900). **f** Violin plots comparing the number of unique contacts per cell among dscHi-C datasets and published scHi-C datasets. **g** Schematics comparing the cell throughput and experimental duration among four high-throughput scHi-C techniques. For non-droplet techniques, the throughput was estimated between 1 and 10 plates per batch. **h** dscHi-C delineates cell types. Left: Uniform manifold approximation (UMAP) showing the embedding of four human cell lines of dscHi-C data. Right: Heatmap showing the scA/B values (calculated by Higashi) of cell-type-specific marker genes.

Here we repurpose a commercially available droplet microfluidics platform to devise a high-throughput scHi-C assay (Fig. 1a, see the section “Materials and methods”). Notably, this approach obviates the need for customized devices, reagents, or oligonucleotides, and can profile tens of thousands of single cells in a single batch, and significantly shortens the sample preparation time. Recently, a similar droplet-based Hi-C assay was reported^25^, which represents an important advancement in the field. However, it faces challenges in detection efficiency, which may limit its ability to reliably identify cell types directly from chromatin architecture features (see detailed discussion below).

## Results

### Development of a high-throughput droplet-based scHi-C technique

Unlike plate-based scHi-C, which decrosslinks crosslinked nuclei that were processed through chromosome conformation capture (3C) before Tn5 tagmentation, droplet-based single-cell library preparation requires nuclei to remain intact prior to droplet emulsification. Consequently, in situ, tagmentation of Hi-C-proceeded nuclei is necessary to ensure compatibility with the droplet-based system. Additionally, amplification of the tagmented chromatin without decrosslinking is required. Initially, we assessed Tn5 tagmentation on nuclei processed using the conventional Hi-C procedure^26^, employing a single restriction enzyme (RE) that resulted in long ligation products (5.1 kb). However, we found that the bulk tagmented DNA fragments were too lengthy for effective amplification (Supplementary Fig. S1a, b). To address this issue, we adopted the Hi-C 3.0 procedure^27^, which utilizes two distinct REs simultaneously and observed a successful in situ fragmentation of chromatin into amplifiable DNA fragments (Supplementary Fig. S1c, d). Furthermore, we demonstrated the feasibility of amplifying these DNA fragments without the need for decrosslinking the tagmented nuclei (Supplementary Fig. S1e). These experiments collectively demonstrate that droplet-based scHi-C library preparation is achievable.

We first validated the droplet single-cell Hi-C (dscHi-C) approach on a mixture of human B lymphoblastoid cells (GM12878) and mouse embryonic stem cells (mESCs). Briefly, freshly harvested cells were crosslinked with paraformaldehyde (PFA) and disuccinimidyl glutarate (DSG), then proceeded using the Hi-C 3.0 procedure, followed by further sodium dodecyl sulfate (SDS) treatment designed to remove open chromatin bias and increase Tn5 tagmentation efficiency. Then nuclei were bulk transposed with Tn5 transposase then loaded on 10x chromium droplet platform for cell barcoding (Fig. 1a, see the “Materials and methods” section). We termed this assay droplet dscHi-C.

To process dscHi-C data, we developed a dscHi-C tool package (see the “Materials and methods” section), designed to execute cell calling, mapping, contact identification and file generation for downstream scHi-C analysis packages like Higashi^28^. The data successfully separated human and mouse cells with low collision and cross-contamination rates (Fig. 1b), demonstrating the feasibility of dscHi-C.

Subsequent verification on mESC cells yielded 223 million contacts, encompassing both intra-chromosomal (> 1 kb) and inter-chromosomal interactions, from ~17,000 single cells across two reactions. The median number of contacts per cell was ~20k and 10k, with duplication rates of 43% and 30%, respectively (Supplementary Table S1). Upon examination of genome coverage, we found that no significant open chromatin bias was introduced during bulk Tn5 transposition (Supplementary Fig. S2a–c). Ensemble dscHi-C profiles accurately recapitulated multi-scale chromatin structures from compartments to topologically associated domains (TADs) and loops, consistent with the observations from bulk Hi-C data (Fig. 1c, d; Supplementary Fig. S2d–f).

We then conducted deep sequencing on subsampled droplets to estimate the library complexity of dscHi-C, revealing that it achieves > 100k unique contacts per cell at a sequencing depth of 1 million reads (Fig. 1e). Comparison with published scHi-C datasets demonstrated impressive throughput and sensitivity of dscHi-C, with comparable intra-chromosomal contacts (Fig. 1f; Supplementary Fig. S2g, h and Table S2). Compared to plate-based scHi-C methods^1^, combinatorial indexing-based sciHi-C, and s3-GCC, dscHi-C achieved the highest throughput with significantly reduced labor and time requirements (Fig. 1g).

### dscHi-C resolves cell identity, copy number, and structural variations

Subsequent application of dscHi-C to a mixture of human cell lines (K562, eHAP, HCT-116, and BJ) facilitated robust delineation of cell identity, highly consistent with the identity based on single nucleotide polymorphisms (SNPs) (Fig. 1h). Moreover, dscHi-C profiles precisely depicted cell-type-specific chromatin structural features (Fig. 1h).

In addition to providing chromatin structure information, dscHi-C serves as a low-depth whole-genome sequencing tool, which is particularly valuable for copy number variation (CNV) studies. Given our cell line mixture contains both euploidy and aneuploidy cells, our findings underscored dscHi-C’s effectiveness in detecting CNV at single-cell resolution (Supplementary Fig. S3a, b). Furthermore, leveraging Hi-C profiles as an effective method to identify structural variations (SVs)^29^, we successfully demonstrated that dscHi-C profiles reliably captured known SVs within these cell lines (Supplementary Fig. S3c). Such analytical capabilities hold significant promise for clinical tumor samples characterized by complex CNVs and SVs.

Chromatin structure undergoes extensive reorganization throughout the cell cycle^30^, a process elucidated using scHi-C maps for computational cell cycle phasing^8^. Here, we illustrated that dscHi-C single-cell profiles effectively captured the continuous transition of chromatin structure during the cell cycle and the characteristic chromatin reorganization (Supplementary Fig. S4). Collectively, we successfully developed a robust and user-friendly high-throughput scHi-C technique, dscHi-C, which not only offers 3D genome structure information but also detects CNVs and SVs.

### dscHi-C reveals cell type-specific chromatin structures in mouse brain

**Fig. 2:**
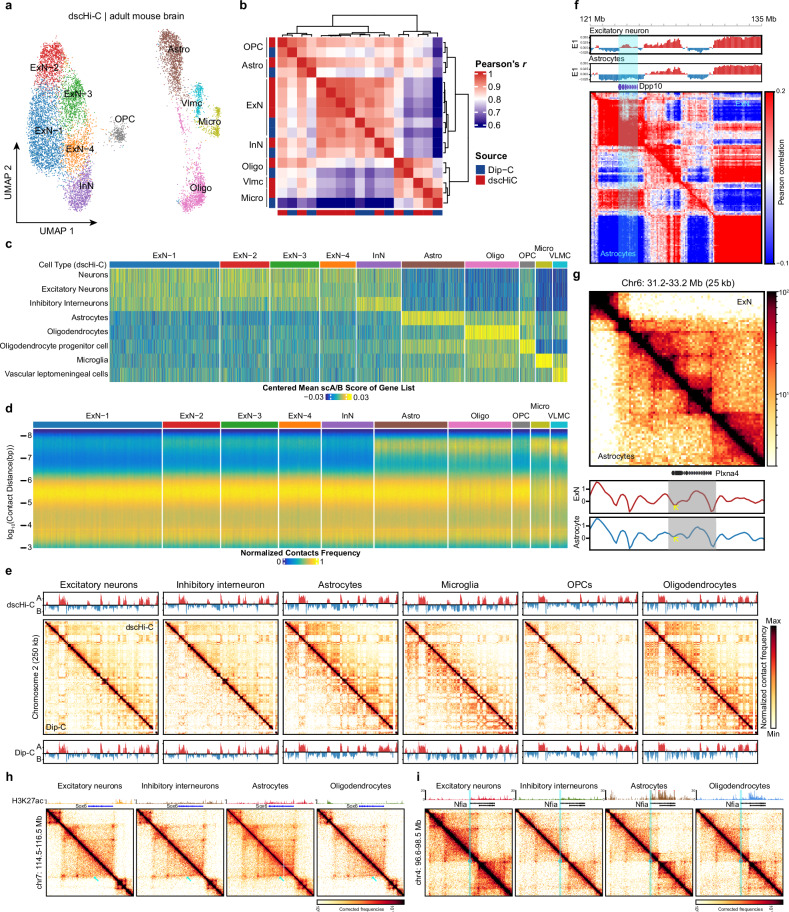
Cell type-specific chromatin structures in adult mouse brain revealed by dscHi-C. **a** UMAP embedding of cell types in adult mouse brains generated from dscHi-C data, the embedding was conducted by Fast-Higashi. **b** Heatmap showing the pairwise correlations of eigenvalues across each cluster, Dip-C data were incorporated. **c** Heatmap showing the normalized scA/B values of marker genes corresponding to each cell type, the marker gene list was obtained from ref. ^1^. **d** Heatmap illustrating the distribution of contact frequency vs. genomic distance. **e** Contact maps of chromosome 2 at 250-kb resolution, Dip-C data were plotted at the lower left, and dscHi-C data were plotted at the upper right. **f** Pearson correlation matrix at *Dpp10* locus at 250-kb resolution. Eigenvalues were plotted on the top. **g** Representative contact map at *Plxna4* locus showing the increased insulation score in ExN compared to Astro. **h** Contact maps at *Sox6* locus, showing the gene stripe structure in InN and Astro. The H3K27ac track was plotted on the top. **i** Contact maps at *Nfia* locus, depicting the enhancer–promoter loop in Astro.

To demonstrate the feasibility of dscHi-C in elucidating cell type-specific chromatin structures within complex tissues, we applied dscHi-C to the adult mouse brain, which has been comprehensively profiled previously^1^. We profiled 10,118 cells from the cortex of 3-month-old mice, yielding a median of 89,883 unique contacts, which is approximately three times the number of contacts reported in recent droplet Hi-C datasets of the mouse brain (Fig. 1f). Based on their chromatin structure profiles, we found that dscHi-C effectively distinguished seven major cell types: excitatory neurons (ExN), inhibitory neurons (InN), oligodendrocyte precursor cells (OPC), oligodendrocytes (Oligo), astrocytes (Astro), vascular leptomeningeal cells (VLMC) and microglia (Micro) (Fig. 2a). Importantly, the cell type classifications obtained from dscHi-C were consistent between FastHigashi and scA/B values defined by Dip-C package (Supplementary Fig. S5a, b), indicating the robustness of cell type identification. For comparison, we reanalyzed droplet Hi-C data from the mouse brain^25^ with the same pipeline and found that the major cell types were not effectively resolved with either FastHigashi or scA/B value in the absence of data imputation (Supplementary Fig. S5c–f), likely due to the sparsity of the data.

To further validate the capability of dscHi-C in accurately capturing cell type-specific chromatin structures, we compared our findings with existing datasets derived from high-resolution Dip-C profiling of the mouse brain. Our results indicated a strong concordance between cell types resolved by dscHi-C and those identified by Dip-C (Fig. 2b). Additionally, the analysis of marker gene expression corroborated that dscHi-C effectively captured distinct chromatin structures associated with specific cell types (Fig. 2c). Consistent with prior reports^1^, we observed that glial cells exhibited more prominent long-range chromatin interactions compared to neurons (Fig. 2d). Detailed examination of the contact maps further revealed a high degree of agreement between dscHi-C and Dip-C data (Fig. 2e).

Moreover, our results demonstrated that dscHi-C accurately reflects the known correlations between chromatin structure and gene expression, including compartmentalization and insulation scores (Fig. 2f, g; Supplementary Fig. S5g, h). At a finer scale, we identified that highly expressed long genes form “promoter stripe” structures within their corresponding cell types (Fig. 2h), which is reminiscent of our observation in GM12878^31^. Additionally, we identified cell type-specific chromatin loops associated with enhancers and promoters (Fig. 2i). Collectively, these results illustrate that dscHi-C can effectively resolve cell types within complex tissues and elucidate the relationship between chromatin architecture and gene expression.

### Dynamic chromatin structure changes during mouse brain aging

**Fig. 3:**
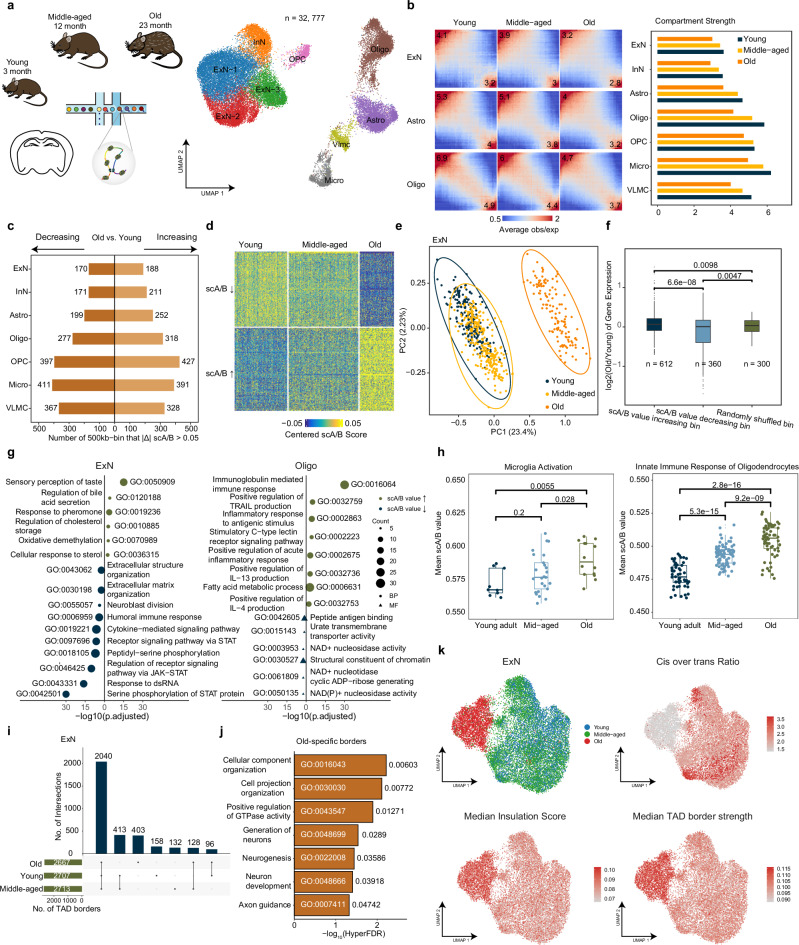
Dynamic changes of chromatin compartments and TADs during mouse brain aging. **a** Schematic overview of experimental design and sampling time points. **b** Saddle plot of different cell types during aging (upper). Visualization of compartment changes at the given locus (below). Old samples are depicted in the upper-right panel while young samples are in the lower-left panel. **c** Summary of 500 kb-bins with a difference of scA/B values > 0.05 in different cell types across aging. **d** Heatmap of centered scA/B values of dynamic 500 kb-bins in ExN. **e** PCA plot of scA/B values of dynamic 500 kb-bins in ExN. **f** Box plot of gene expression changes in scA/B value increased, decreased, and randomly shuffled bins comparing old to young in microglia. **g** Pathway enrichment analysis of genes located within aging-related bins. **h** Comparison of mean scA/B values in microglia activation and innate immune response gene modules of microglia and oligodendrocytes across aging. **i** Summary of TAD borders of ExN across aging. **j** GREAT analysis of old-specific TAD borders (10 kb) in ExN. **k** UMAP embedding of excitatory neurons. Colored by the ratio of intra-chromosomal vs. inter-chromosomal interactions, TAD insulation score, and TAD strength.

Chromatin structures undergo extensive reorganization throughout brain development and aging^11,32^. However, the remodeling of chromatin architecture during the aging of the mouse cerebrum remains unexplored. To investigate this, we further conducted high-throughput dscHi-C on samples from the mouse brain cortex at two distinct stages: 12 months (middle-aged) and 23 months (aged). Alongside data from 3-month-old mice, we profiled a total of 32,777 single cells, yielding a median of 78,220 unique contacts (Fig. 3a).

We subsequently integrated these three datasets to analyze alterations in chromatin structure associated with aging. Our initial examination of large-scale chromatin organization revealed a global weakening of compartmentalization across all cell types, indicating a mixing of A/B compartments (Fig. 3b; Supplementary Fig. S6a, b). Notably, we observed significant alterations in contact frequency relative to genomic distance in aged mice, characterized by a marked decrease in short-range interactions and an increase in long-range interactions (Supplementary Fig. S6c). This pattern exhibited two distinct behaviors between neurons and OPC vs. glial cells. Cells exhibited a similar pattern in middle-aged and young mice across all cell types, indicating no significant change in chromatin architecture between these two stages.

To alleviate the scHi-C data sparsity, we aggregated single cells into metacells for further analysis (Supplementary Fig. S7a, b), achieving a median of ~2 million contacts per metacell. The metacells exhibited clear distinctions between various cell types, along with more pronounced cell type-specific features (Supplementary Fig. S7c–e).

We defined genomic regions exhibiting significant compartment changes based on metacell classifications (Fig. 3c, d). Our analysis indicated that non-neuronal cell types displayed a greater number of age-related genomic regions (Fig. 3c), consistent with the observations that glial cells exhibit more differentially expressed genes compared to neurons^20^. Principal component analysis (PCA) of differential scA/B values distinctly separated metacells of aged mice from those of young and middle-aged mice (Fig. 3e). This finding, combined with the distribution of contact distance, suggests that the remodeling of 3D genome architecture does not occur gradually during mouse brain aging. Furthermore, when we examined single-cell expression data from aging mice^20^, we found that the observed compartment changes aligned with trends in gene expression alterations (Fig. 3f).

Subsequently, we analyzed the functions of genes within genomic regions that exhibited altered scA/B values. We found that genes with increasing scA/B values were enriched in pathways associated with sensory perception, pheromone response, and oxidative demethylation in neurons. In contrast, non-neuronal cell types consistently demonstrated enrichment in pathways related to the innate immune response, such as inflammatory response, cytokine production (e.g., IL-4 and IL-13), antigen presentation, and chemokine binding (Fig. 3g; Supplementary Fig. S8c, d). Conversely, genes located in bins with decreasing scA/B values were enriched in pathways related to extracellular matrix organization and metabolic processes, including NAD(P)+ nucleosidase activity across all cell types. Notably, the cytokine-mediated signaling pathway (e.g., JAK-STAT pathway) was exclusively enriched in ExN, highlighting a distinct impact on these cells during aging. Given that chronic inflammation is a common hallmark of aging^17,33^, we examined how compartments change for genes involved in inflammation and found a significant increase in scA/B values (Fig. 3h; Supplementary Fig. S8e), indicating their activation^20^.

Domain analysis of ExN pseudobulk revealed that ~78% of TAD borders (10 kb) were shared across three ages (Fig. 3i). GREAT analysis of old-specific TAD borders in ExN showed enrichment in pathways related to morphological changes (cell projection organization and axon guidance), neurogenesis and neuron development (Fig. 3i, j). To determine whether the gene body (gene length > 300 kb) underwent TAD establishment or melting during aging, we employed the “MELTRON” pipeline^34^. Similarly, we found that the number of changed genes was higher in non-neuronal cell types than in neurons (Supplementary Fig. S9a). Genes that underwent domain melting such as *Esrrg*, were up-regulated during aging, while genes that established domains, such as *Plce1*, were down-regulated accordingly (Supplementary Fig. S9b). This suggests that reformed domains can mediate genomic regulatory elements that activate or promote transcriptional processes.

To test whether cells from aged mice can be distinguished solely by chromatin architecture, we extracted the annotated ExN cells and re-embedded into a low-dimensional representation space (see Materials and methods). Briefly, we focused on ExNs that have sufficient cell numbers and found that ExN cells from aged mice clearly formed a distinct cluster. This clustering was primarily dominated by increased inter-chromosomal intermingling and enhanced TAD structures (Fig. 3k; Supplementary Fig. S10). Collectively, these findings highlight the chromatin reorganization that occurs during aging in the mouse brain, characterized by enhanced mixing of the A/B compartment, increased inter-chromosomal and long-range interactions, and a reinforcement of TAD structures.

### Droplet-based single-cell joint chromatin structure and gene expression assay

**Fig. 4:**
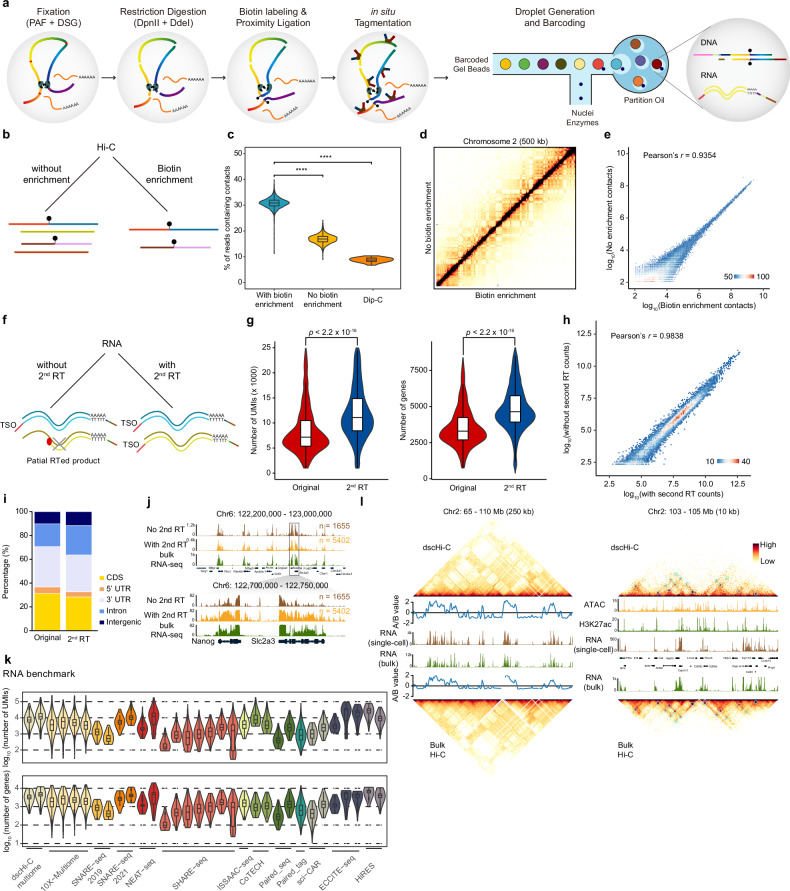
Droplet-based single-cell joint Hi-C and gene expression co-assay. **a** Schematics of the droplet single-cell simultaneous Hi-C and RNA profiling method. **b** Schematic showing two Hi-C part processing procedures, with and without biotin enrichment. **c** Violin plot comparing the percentage of reads containing contacts for with and without biotin enrichment, Dip-C was plotted for comparison. **d** Contact map of with (bottom right) and without (top left) biotin enrichment of chromosome 2 at 500-kb resolution. **e** Density plot showing the correlation of raw Hi-C contact counts at 500-kb resolution between with and without biotin enrichment. **f** Schematic showing two RNA part processing procedures, with and without second RT. **g** Violin plot comparing the scRNA-seq library size (left) and the number of genes (right) with and without second RT. **h** Density plot showing the correlation of raw RNA counts for each gene between with and without second RT. **i** RNA reads the composition of with and without second RT RNA libraries. **j** Aggregated scRNA-seq profiles along with bulk RNA-seq of mESC at *Nanog* genomic loci. **k** Benchmark of scRNA-seq library size (top) and the number of detected genes (bottom) between dscHi-C multiome and other published single-cell multi-omics datasets. **l** Contact maps of ensemble dscHi-C multiome and bulk Hi-C accompanied with eigenvalue, RNA-seq track, ATAC-seq, and H3K27ac tracks.

Understanding the intricate relationship between genome structure and gene regulation necessitates concurrent genome structure and gene expression assays. While several related co-assays have been developed recently^24,35,36^, they are labor-intensive and require custom reagents and oligos. Here, we incorporated gene expression detection into the dscHi-C assay, creating a droplet-based single-cell multi-omics assay, termed dscHi-C multiome (Fig. 4a, see the “Materials and methods” section).

For the Hi-C part of dscHi-C multiome, we employed biotin enrichment to increase the percentage of reads containing contacts (referred to as contact ratio) (Fig. 4b). As expected, the use of biotin enrichment substantially elevated the contact ratio compared to the procedure without enrichment, resulting in a contact ratio of 30.9% vs. 16.7% (Fig. 4c). Moreover, biotin enrichment enabled the detection of a comparable number of contacts (27k vs. 33k) using fewer sequencing reads (218k vs. 514k) (Fig. 1f). Notably, the resulting contact profiles showed no significant difference between data with and without biotin enrichment (Fig. 4d, e), and they exhibited a high degree of correlation with dscHi-C only and bulk Hi-C datasets (Supplementary Fig. S11a–d).

Regarding the RNA part of dscHi-C multiome, we explored a second reverse transcription (RT) treatment after droplet pooling and decrosslinking. This exploration is motivated by the fact that many RNA molecules are crosslinked with proteins, which impedes reverse transcriptase from synthesizing full-length transcripts (Fig. 4f). Indeed, the additional RT treatment significantly enhanced RNA capture efficiency (genes: 4640 vs. 3294; unique molecular identifiers (UMIs): 11,860 vs. 7369) (Fig. 4g) without compromising data quality (Fig. 4h, j). The resulting scRNA-seq gene expression profiles demonstrate high consistency among themselves and with bulk RNA-seq data (Fig. 4j; Supplementary Fig. S11e, f). When compared with published single-cell multi-omics datasets, dscHi-C multiome RNA detection efficiency outperforms most published datasets (Fig. 4k).

The simultaneous measurement of chromatin structure and gene expression is crucial for studying the chromatin architecture underlying cell-type-specific gene regulation in complex tissues. We showed that dscHi-C multiome effectively captures both expression status and chromatin structure with high precision (Fig. 4l).

### Chromatin structures behind cell type-specific gene expression in mouse brain by dscHiC-multiome

**Fig. 5:**
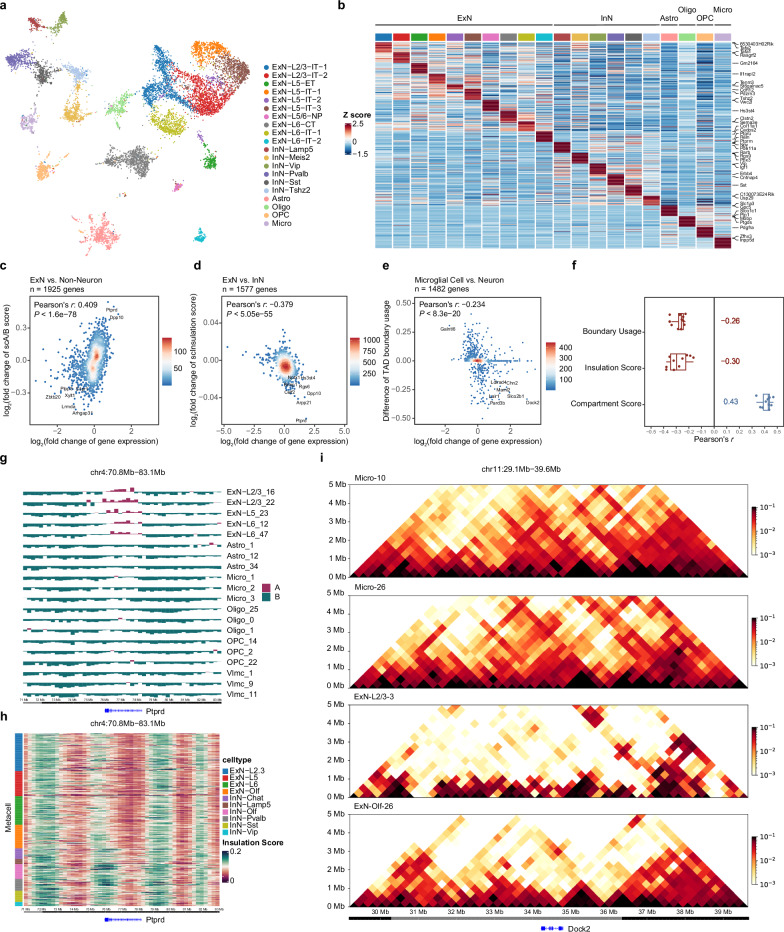
Droplet-based single-cell joint chromatin structure and gene expression co-assay of mouse brain. **a** UMAP visualization of annotated single-cell clusters of mouse brain. **b** The expression patterns of cell-type-specific marker genes. **c** Correlation between gene expression change and scA/B value change in ExN compared to non-neuron. Pearson correlation co-efficient is calculated. **d** Correlation between gene expression change and insulation score change in ExN compared to InN. **e** Correlation between gene expression change and TAD boundary usage change in microglia compared to neurons. TAD boundary usage was defined as boundary frequency in cells of the same cluster. **f** Box plot depicting the correlation between different metrics and gene expression (compartment score, insulation score, TAD boundary usage) of different comparisons (*n* = 10, e.g. InN-Sst vs. InN, ExN vs. non-neuron and so on). **g** Visualization of scA/B values in *Ptprd* gene region across ExN and non-neuron. Each row is a randomly chosen metacell. **h** Heatmap of insulation scores in *Ptprd* gene region across ExN and InN. **i** HiC interaction heatmap of *Dock2* locus across microglia and ExN metacells.

To illustrate the capabilities of dscHiC-multiome, we sequenced 10,087 single cells from the brain cortex of young mice, resulting in a median of 5191 contacts, 1250 detected genes, and 1968 UMIs. The clustering analysis of RNA data identified 20 sub-clusters (Fig. 5a), indicating that the integration of RNA profiling captures cell types with greater resolution compared to dscHiC data alone. Layer-specific ExN subtypes were also identified by marker genes such as *Tafa2*, *Il1rapl2*, and so on (Fig. 5b). InN were classified by uniquely expressed markers like *Lamp5*, *Meis2*, *Vip*, *Pvalb*, *Sst*, and *Tshz2*. Additionally, non-neuronal cell types, including Astro, Oligo, OPC, and Micro were also identified. However, VLMC was not detected, likely due to the limited number of the cells profiled.

Recent studies have made significant efforts to explore how 3D genome organization influences transcriptional regulation^35,37^. To quantitively assess the relationship between multiscale 3D genome organization and gene expression, we re-calculated scA/B value, insulation score, and TAD boundary at the metacell level defined by RNA clusters (Supplementary Fig. S12a, b), aiming to minimize potential noise in scHiC data. Before downstream analysis, we removed potential doublets based on RNA expression profiles (Supplementary Fig. S12c–e). We observed a strong correlation between the expression of ExN-specific genes (*n* = 1925) and scA/B values when comparing ExN to non-neuronal cell types (Fig. 5c). Several ExN marker genes, such as *Ptprd* and *Dpp10*, exhibited significant correlations. Visualization of the compartmentalization of the *Ptprd* gene body within metacells confirmed that *Ptprd* resides in the A compartment, maintaining high expression in ExN, while it is located in the B compartment in non-neuronal cell types (Fig. 5g).

We further investigated the relationship between insulation scores at gene bodies and gene expression, revealing an expected negative correlation (Fig. 5d). Highly expressed marker genes in ExN, compared to InN, displayed low insulation scores, exemplified by the *Ptprd* gene (Fig. 5h). Changes in TAD boundaries may create distinct domains that facilitate interactions between gene regulatory elements. Additionally, we explored whether TAD boundary usage, defined by the frequency of boundary overlap with transcription start sites (TSS) across cell populations, correlates with gene expression profiles. A weak negative correlation was observed for genes expressed in microglia compared to neuronal cell types (Fig. 5i). However, some highly expressed marker genes exhibited significantly low boundary usage. For instance, stronger interaction strength was observed in microglia metacells surrounding the TSS and gene body of *Dock2*, whereas ExN metacells displayed a clear boundary at the TSS, interrupting interactions between the upstream region of the TSS and the gene body. To validate that these correlations are not cell type-specific, we repeated the analysis across multiple comparisons (*n* = 10) among various main sub-cell types. The results indicated consistent correlations across all cell types, although the correlation of insulation scores exhibited greater variability (Fig. 5f). This evidence suggests that different gene sets may exhibit unique patterns in response to changes in TAD domains, with ExN marker genes showing the strongest correlation with TAD boundary usage (*R* = –0.379), while Oligo marker genes compared to neuronal cells demonstrated the weakest correlation (*R* = –0.164).

## Discussion

The development of scHi-C technique has lagged behind other single-cell genomics technologies, such as scRNA-seq, scATAC-seq, and scCUT&Tag, primarily due to throughput and cost hurdles. These aforementioned techniques have been successfully implemented on droplet-based platforms and have emerged as powerful tools for deciphering gene regulatory programs in both developmental processes and disease contexts^38–43^. The implementation of dscHi-C technique fills this gap in high-throughput 3D genome analysis within droplet-based platform. Its application to cell atlas study adds critical 3D interaction regulatory information beyond identifying *cis*-regulatory elements. Additionally, dscHi-C offers simultaneous CNV and SV analysis capabilities, making it a valuable tool for various applications, particularly in investigating the heterogeneity of tumor subclones and elucidating mechanisms underlying tumorigenesis in clinical tumor samples.

Our methods strive to balance throughput and detectability, both of which are essential for establishing a widely applicable technology. The high-throughput advantages of droplet microfluidics allow researchers to conduct large-scale cell atlas studies in development and disease more cost-effectively. More importantly, the enhanced sensitivity of dscHi-C enables the detection of genuine biological variation at single-cell resolution, significantly decreasing sparsity compared to previous high-throughput single-cell Hi-C methods^14^. Despite the rapid advancement of scHi-C imputation software, our approach’s superior detectability minimizes the artificial biases that may arise during imputation, enhancing the reliability of downstream analyses.

Significant epigenetic changes occur during the aging process, including reductions in global heterochromatin, nucleosome reorganization, and alterations in histone modifications and DNA methylation^44^. Notably, a DNA methylation-based epigenetic clock has been proposed as a measure of biological age^45^. However, the exploration of chromatin architecture in this context remains limited. The application of dscHi-C to aged mouse brains has revealed extensive rewiring of large-scale chromatin interactions during brain aging and its correlation with gene expression changes. Our data indicate a decrease in compartment strength and an enhancement of TAD structures during mouse brain aging, suggesting both global and local chromatin remodeling in response to intrinsic or extrinsic stimuli. Genes associated with genomic bins exhibiting compartment shifts or age-specific TAD borders were significantly enriched in pathways related to immune response, metabolic processes, and morphological changes, corroborating findings from other studies on transcriptional profiles^46–48^. This evidence suggests a coordinated pattern between chromatin remodeling and transcriptional changes, integrating data from other snRNA-seq datasets. However, due to the limited datasets generated in this study, we could not explore changes in fine-scale chromatin structures, such as chromatin loops. Future research should focus on generating larger datasets alongside high-resolution single-cell 3D genome data from techniques like Dip-C or scMicro-C.

## Materials and methods

### Mice

The study was approved by the Peking University Laboratory Animal Research Center Institutional Animal Care and Use Committee (IACUC), and all animal experiments were conducted following the ethical guidelines. Mouse brain samples were taken from the F1 hybrids of CAST/EiJ (JAX 000928)×C57BL/6J (JAX 000664). For each stage (3 months, 12 months, and 23 months), one female and one male mouse were used.

### Cell culture

K562 cells (ATCC, CCL-243) were cultured in Iscove’s modified Dulbecco’s medium. BJ cells (ATCC, CRL-2522) were grown in ATCC-formulated Eagle’s minimum essential medium. eHAP cells (Cellosaurus) and engineered haploid chronic myeloid leukemia cells were grown in Iscove’s modified Dulbecco’s medium. GM12878 cells (Coriell Institute) and B lymphoblastoid cells were grown in Roswell Park Memorial Institute 1640 Medium. These media were supplemented with 10% FBS and 1% Penicillin/Streptomycin, except for GM12878 supplemented with 15% FBS.

mESC were grown in knockout DMEM medium containing 15% FBS, 1% Penicillin/Streptomycin, 2 mM l-glutamine, 1 mM non-essential amino acids, 1× nucleosides, 3 μM CHIR99021, 1 μM PD0325901, 0.1 mM 2-mercaptoethanol and 1000 U/mL LIF. The plate was pretreated with 1% gelatin. All cells were maintained at 37 °C with 5% CO_2_ at recommended density.

### Mouse brain nucleus isolation and crosslinking

The mice were euthanized using CO_2_, after which their brains were extracted. The brain cortices were dissected on a dish containing ice-cold PBS and then homogenized using a Dounce homogenizer as previously described^1^. Briefly, the brain cortices were minced, then transferred to a precooled 2-mL Dounce homogenizer containing 2-mL nuclei isolation buffer (0.25 M sucrose, 25 mM KCl, 5 mM MgCl_2_, 10 mM HEPES pH 8.0, 1 μM DTT, 0.1% Triton X-100), first by 15 strokes with the loose pestle A, followed by 15 strokes with the tight pestle B. Then the samples were transferred to a precooled 5-mL tube, centrifuged at 100× *g* for 8 min using a centrifuge precooled to 4 °C with a swing bucket, washed twice with 4 mL of nuclei wash buffer (0.25 M sucrose, 25 mM KCl, 5 mM MgCl_2_, 10 mM HEPES pH 8.0, 1 μM DTT) and filtered through a 30-μm cell strainer. Then the nuclei were crosslinked with 1% PFA, followed by 3 mM DSG, aliquoted, and stored at –80 °C.

### Hi-C 3.0 procedure

The Hi-C 3.0 experiments were performed as previously described^49^. Briefly, cells were firstly double crosslinked with PFA followed by DSG, then treated with 0.5% SDS and Triton X-100. Then the chromatin was digested by resuspending the nuclei in a digestion mix containing DdeI and DpnII without detergent, and incubated at 37 °C for 2 h, followed by inactivating the REs. The DNA ends were filled by biotin-labeled dATP, followed by proximity ligation using T4 ligase by incubating at room temperature for 2 h. For samples prepared for the dscHi-C multiome experiment, all reaction mixtures were supplemented with 0.5 U/μL Protector RNase inhibitor.

### dscHi-C procedure

The dscHi-C samples were proceeded with 10x ATAC kit v2. The Hi-C 3.0-proceeded nuclei were further treated with 0.1% SDS by incubating at 55 °C for 1 h, then quenched by 1% Triton. This treatment was estimated to cause ~20% of nucleus loss. Then nuclei were filtered with a 10-μm strainer, then 20,000 nuclei were aliquoted as input and resuspended in 5 μL of 1× Nuclei Buffer (10x ATAC kit v2). Then the nuclei were proceeded with “CG000496_Chromium_NextGEM_SingleCell_ATAC_ReagentKits_v2_UserGuide_RevB.pdf” with slight modifications. Briefly, the bulk transposition was incubated at 37 °C for 1 h followed by 55 °C for 30 min. Then the reaction was stopped by adding 24 μL of 20 mM EDTA containing 0.1% SDS to stop the reaction by incubating at 37 °C for 10 min, then quenched by 1% Triton. Transposed nuclei were further filtered with a 10-μm strainer to remove multiplets. Then the nuclei were resuspended in 8 μL 1× Nuclei Buffer and 7 μL ATAC buffer B, then loaded on the 10x chip H. The GEM was collected, and the linear amplification was modified to prolong the extension time from 1 to 2 min. After GEM incubation, the nuclei were decrosslinked by adding 5 μL QIAGEN proteaseK and incubating at 65 °C for 1 h. Then the purified linear amplified DNA product was subjected to library preparation, the amplification was performed as follows: 72 °C, 5 min; 98 °C, 30 s; 5–8 cycles of [98 °C, 10 s; 67 °C, 30 s; 72 °C; 2 min]; 72 °C, 2 min. Then the library was purified with 1.6× SPRI bead followed by 0.8× SPRI bead selection to remove short DNA fragments. Then the dscHi-C library was sequenced on Illumina Nova6000 S4 or NovaSeq X Plus platform. 7 dscHi-C reactions were generated in this study: GM12878-mESC mixture, mESC reaction 1, mESC reaction 2, mESC subsample was taken from a subset of a droplet of mESC reaction 1, and human cell line mixture, mouse brain cortex samples collected from 3-month-old, 12-mon-old, and 23-month-old mice. The information on these libraries was recorded in Supplementary Table S1.

### Difference between dscHi-C and droplet Hi-C

The dscHi-C assay differs from the recently published droplet Hi-C assay^25^ in several key aspects. First, dscHi-C utilizes dual crosslinking with PFA and DSG, whereas droplet Hi-C relies solely on PFA. Notably, previous studies have reported that dual crosslinking can reduce background noise in chromatin interaction data^27^. Second, dscHi-C employs two restriction enzymes followed by an end-repair step to generate blunt ends, while droplet Hi-C uses three restriction enzymes (DpnII and MboI, which recognize the same sites) without an end-repair step. Finally, dscHi-C incorporates an additional SDS treatment step to minimize bias toward open chromatin regions, a step that was not included in the droplet Hi-C protocol.

### dscHi-C multiome procedure

The dscHi-C multiome sample proceeded with 10x multiome ATAC + gene expression kit. The Hi-C 3.0 procedure was slightly modified. Specifically, we adjusted the SDS concentration to 0.3% to retain more RNAs and supplemented with RNase inhibitor at 1 U/μL in all reaction buffers. And no further SDS treatment was performed. The Hi-C 3.0-processed nuclei were filtered with a 10-μm strainer, counted, and aliquoted to provide 20,000 nuclei as input, then resuspended in 5 μL of 1× Nuclei Buffer (10x multiome). Then the nuclei were proceeded with “CG000338_ChromiumNextGEM_Multiome_ATAC_GEX_User_Guide_RevF.pdf” with some modifications. Briefly, the nuclei were transposed at 55 °C for 20 min. The transposed nuclei were loaded on 10x chip J. Post GEM incubation, the samples were decrosslinked by adding 5 μL QIAGEN proteaseK and incubating at 65 °C for 1 h.

#### Biotin enrichment procedure

The purified sample was subjected to Streptavidin bead pull down (Hi-C DNA) and the supernatant (RNA) was collected and transferred to a new tube and purified with 1.6× SPRI beads. The DNA was directly subjected to library preparation on Streptavidin beads by incubating as follows: 72 °C, 5 min; 98 °C, 30 s; 12–14 cycles of [98 °C, 10 s; 67 °C, 30 s; 72 °C; 2 min]; 72 °C, 2 min]. The paired RNA was subjected to a second RT treatment (see below).

#### Without biotin enrichment procedure

The purified sample was subjected to preamplification, then split into two parts, and the DNA parts was amplified as follows: 98 °C, 45 s; 5–7 cycles of [98 °C, 10 s; 67 °C, 30 s; 72 °C; 2 min]; 72 °C, 2 min. The paired RNA was proceeded to further amplification and library preparation according to the manual without second RT.

#### Second RT procedure

The purified sample was subjected to a second RT reaction performed in 100-μL RT mix (1× RT Buffer, 0.5 mM dNTP, 8 mM DTT, 5% PEG8000, 2.5 mM MgCl_2_, 0.5 U/μL RNase inhibitor, 25 U/μL Maxima H-minus RTase, 2 μL 10× Template Switch Oligo), incubated as follows: 42 °C, 30 min; 10 cycles of [50 °C, 2 min; 42 °C, 2 min], 85 °C, 5 min. Then, the product was purified with 1.6× SPRI beads and then subjected to preamplification and library preparation according to the manual.

#### Sequencing

The dscHi-C multiome DNA library and RNA library was sequenced on Illumina Nova6000 S4 or NovaSeq X Plus platform. The RNA was sequenced at 50k reads per cell, the DNA library was sequenced at 300k reads per cell.

### DNA fragment length distribution quantification

The DNA fragment length distribution was quantified through capillary electrophoresis by a Fragment Analyzer with DNF 474 NGS kit. The data were exported by Prosize software (v3.0) and visualized by Python matplotlib.

### dscHiC data preprocessing

To make dscHiC data compatible with downstream analysis, we developed an ensembled tool called “dscHiCtools” (https://github.com/MaoxuWang/dscHiCtools). dscHiCtools firstly identified the true cell barcodes from index reads against whitelist (cellranger-atac-2.1.0/lib/python/atac/barcodes/737K-cratac-v1.txt.gz) barcodes that allow one mismatch. Barcodes with no or multiple hits will be discarded. Called cell barcodes were added to read the name. Reads then were mapped to reference genome by bwa-mem^50^(v 0.7.17) in 5SP mode. Hickit was used to extract and dedup contacts. Notably, we did minor modifications to Hickit (https://github.com/MaoxuWang/hickit) to adapt the single-cell features. Briefly, we extracted the cell barcode to each contact information, and the cell barcode was also considered in contact duplication rather than distance (set parameters --dup-dist = 100”) alone. Many of the detected barcodes harbor only a few contacts due to technique noise brought by microfluidic. True cell barcodes were called by threshold. We applied kneedle (R package, v1.0.0) to detect this threshold (turning point) defined by the contact number and its rank of each barcode. To overcome underestimated contact number, we set the parameter “sensitivity” of kneedle function from 1 to 10 and finally selected the one that can more closely retain over 85% of the total contact number.

### Multiplet detection

To remove potential doublet contamination, we used AMULET^51^ (v1.1) to detect multiplets for dscHiC datasets. AMULET is a count-based statistical method that can estimate the probability that a barcode is derived from multiplets. Parameters are set as follows: (“maxinsertsize = 1000”, “start_base” = 0, “end_base” = 0), *q*-value threshold = 0.01). For scRNA datasets, DoubletFinder^52^(v2.0.4) was used with parameters (“pc.num = 1:10”, “pN = 0.25”). The expected multiplet rate was set to 7.5% according to the reference of 10x genomics given the expected cell numbers.

### Cell line cell typing by SNP

To demonstrate the power of cell typing by dscHiC data, we used an independent source-derived cell type label as the golden standard to verify its accuracy. In brief, this external label was achieved by leveraging prior knowledge of heterogenous SNP sites within different cell lines. Souporcell^53^ (v2.5) was performed by setting parameters as follows: “-k = 4, skip_remap = true, no_umi = true” to define the true cell type label for each single cell.

### Bulk RNA-seq data processing

After obtaining bulk RNA-seq data of K562, eHAP, HCT-116, BJ cell line, gene expression quantification was performed by Salmon^54^ (v1.7.0). Count matrix was further imported by tximiport (v1.30.0) to summarize gene-level quantification. The output can be directly loaded into DESeq2^55^ (v1.42) by DESeqDataSetFromTximport function. Cell type specific marker genes were calculated by comparing every one cell type vs the other. Significant differential genes were kept only if its p.adj < 0.001 and |log2FoldChange| > 2.

### Cell-cycle phasing

Prior knowledge was utilized to phase different cell cycles. Metrics for the assignment were defined as Nagano^8^ described.

### dscHi-C multiome HiC data processing

Main pipeline was the same as dscHiC data preprocessing procedure except for the cell barcode calling. Index reads were firstly mapped against a whitelist (cellranger-arc-2.0.2/lib/python/atac/barcodes/737K-arc-v1.txt.gz) and then were converted to RNA barcodes (cellranger-arc-2.0.2/lib/python/cellranger/barcodes/737K-arc-v1.txt.gz). The downstream analysis was also similar to dscHiC data processing.

### dscHi-C multiome RNA data processing

Cell Ranger toolkit (v7.0.1) was applied to align RNA reads and generate a cell-by-gene count matrix using reference genome (“refdata-gex-mm10-2020-A”). Downstream analysis was performed by Seurat^56^(v4.4).

### CNV analysis

HMMcopy^57^ (v1.18.0) was used to profile copy number. To demonstrate the potential power of dscHiC for CNV analysis, we performed this analysis both in single-cell and bulk manner at 1-Mb resolution, with GC and mappability corrected. Segmentation was called by hidden Markov model in HMMcopy.

### Species mixing experiment

Reads from species mixing experiment were mapped to reference genome (refdata-cellranger-atac-GRCh38-and-mm10-2020-A-2.0.0/fasta/genome.fa). Bam file was then split according to chromosome name (“hg38” or mm10) into two bam files to call contacts accordingly. Species ratio was calculated for each cell barcode. Barcodes with a mixing rate (proportion of smaller number of reads that aligned to either hg38 or mm10) > 20% were considered as doublets. Reads with a mapping quality < 20 were discarded.

### Imputation of contact maps with Higashi

To resolve the sparsity of our data, we utilized Higashi^28^ (a hypergraph representation learning-based approach, https://github.com/ma-compbio/Higashi/wiki) to impute dscHiC data. We followed the tutorial to train this model on gpu and saved its output. Parameters were set to default (resolution = 1,000,000 for cell-line and 500,000 for mouse brain libraries).

### Calculation of scA/B compartment score

For higashi-imputed contact maps, we executed “scCompartment.py” in the Higashi tooklit to calculate scA/B compartment score for each cell without neighbor information used. The CpG density generated by “CpG_density.py” was used to calibrate the result. The *z*-score-normalized scA/B values were used in the downstream analysis. For raw single-cell contact maps, we calculated scA/B values as described in Tan et al. using “dscHiCtools scAB” sub-command. Raw scA/B values were used in gene module calculation and imputed scA/B values were used in visualization and inter-sample comparison.

### Calculation of single-cell insulation scores

To accommodate the resolution required for insulation score calculation, we re-trained the higashi model at 250-kb resolution for mouse brain datasets. The single-cell insulation scores were calculated for higashi-imputed contact maps by “scTAD.py” (with parameters “window_ins = 2000000”, “window_tad = 500000”) without neighbor information.

### Joint cell calling in dscHi-C multiome

Single-cell analysis was performed by Seurat (v4.4). We only kept cells with the minimal detection of “> 500 UMIs; > 400 genes; > 1000 contacts pairs; percentage of mitochondrial content < 10%; inter-chromosomal contacts rate < 45%” to the downstream analysis.

### Dimension reduction

For dscHiC datasets, we used Fast-Higashi^58^ to infer a low-dimensional representation of scHiC contact matrix (with parameters “resolution = 250 kb; off_diag = 100; batch_norm = False; do_rwr = True; filter = True; do_conv = False; do_col = False; no_col = False”). Size of the meta-embedding size was set to 256. Finally, we used “embed_l2_norm_correct_coverage_fh” as single-cell low-dimensional representation. The embedding matrix was loaded to create a Seurat object by CreateSeuratObject function of Seurat.

For scRNA datasets in dscHi-C multiome, we firstly normalize count matrix by “NormalizeData” with default parameters. Genes for downstream analysis was selected by FindVariableFeatures (with parameters “selection.method = vst”, “nfeatures = 2000”). Gene expression matrix was further scaled by “ScaleData” (with parameters “vars.to.regress = contactsN”). We further performed PCA by “RunPCA” function in Seurat and retained the first 25 components for downstream analysis.

### Unsupervised clustering

UMAP visualization was performed by “RunUMAP” with default parameters. Unsupervised clustering was performed by “FindNeighbors” (with parameters “dims = 1:25”) and “FindClusters” (with parameters “resolution= seq(0.2, 1,0.2)”).

### Data integration of multiple datasets

To remove the potential batch effect, Harmony^59^ (v0.1) was used on the PCA matrix with default parameters. To note that, UMAP visualization and unsupervised clustering used the corresponding harmony layer instead of original PCA layer.

### Cell type identification

For dscHiC datasets, scA/B value matrix calculated by Higashi was loaded into seurat object by CreateSeuratObject function of Seurat. Top 100 of each cell-type-specific marker genes were selected as gene sets to calculated average scA/B value as Tan (2021) described. In cell-line datasets, cell-type specific marker genes were defined by bulk RNA-seq (see above). In mouse brain datasets, cell-type-specific marker genes were collected in published mouse brain scRNA-seq datasets. Per-single-cell calculation was performed by AddMetaData function. This averaged gene-sets-level scA/B value can be easily visualized or compared and thus determine the cell type information in a cluster-specific manner.

For scRNA datasets of the mouse brain, marker genes were computed by “FindAllMarkers” (with parameters “only.pos = TRUE”, “min.pct = 0.35”, “logfc.threshold = 0.4”). Main clusters were determined at the resolution of 0.2, including ExN, InN, Astro, Oligo, OPC, Micro. To obtain a finer annotation of cell types in mouse brain datasets, we also subtracted the main clusters to do another round of clustering with higher resolution.

### Ensembled contact maps of pseudobulk and metacell

To overcome the intrinsic noise in single-cell HiC data, we aggregated the pseudobulk samples based on main or sub-cell types of unsupervised clustering. Metacells were defined as single cells with similar Hi-C profiles. After annotating each cluster by marker gene list of mean scA/B values, we were able to iteratively cluster single cells within each cell type at a higher resolution (“FindClusters” of Seurat, set the parameters “resolution = 15”), thereby establishing the correspondence of metacells. Single cells belonging to the same cluster were further merged into metacells and processed in the same embedding procedure by FastHigashi. For dscHi-C multiome datasets, metacells were defined as single cells with similar RNA profiles, while the subsequent data processing procedures were consistent with those applied to dscHi-C datasets. Then we merged the contact pair file according to the correspondence. Juicer^60^ (v1.22) “pre” was used to generate.hic file (with parameters “-n; -r 100000,250000,500000,1000000, 2500000” for metacells and “-r 10000,25000,50000,100000,250000,500000,1000000, 2500000” for pseudobulk). Then hic2cool (v0.8.3) was used to convert.hic file to.mcool file. Cooler^61^ (v0.10.2) was used to balance the matrix by iterative correction method. Pseudobulk contact maps were analyzed as standard bulk HiC data processing, while the metacell contact maps were analyzed in a single-cell-like procedure as previously described. Furthermore, we merged the imputed contact maps of metacell by “Merge2Cool.py” of higahsi toolkits for visualization.

### Identification of aging-related compartment change

To define aging-related compartment change, we calculated the median scA/B compartment scores for each cell type of different time points in metacells (old:23 months; middle-aged: 12 months; young: 3 months). Differential analysis was performed by “FindMarkers” specifying the scA/B matrix layer (with parameters “logfc.threshold = 0”, “min.pct = 0”) by Wilcoxon rank sum test. Genomic regions with adjusted *p*-value < 0.01 were retained in the downstream analysis.

To compute averaged scA/B values of the gene module, we extracted gene list from Gene Ontology (GO) database: microglia activation (GO: 1903978), innate immune response (GO: 0045087), astrocytes activation (GO: 0048143), astrocytes migration (GO: 0043615). Averaged scA/B values of the selected gene list of metacels were compared in different time points. A two-sample Wilcoxon rank sum test was then conducted between samples of different time points (old vs young, middle-aged vs. young). Benjamini–Hochberg method was used to adjust *p*-values and adjusted *p*-value < 0.01 was considered as significant.

Domain melting score was calculated on genes longer than 300 kb at 50-kb resolution in pseudobulk samples in a way that was described by Warren Winick-Ng^34^ et al. We performed this procedure by comparing old to young and young to old samples for every cell type. Notably, we define a domain is melted during aging if insulation square values are decreased and established if insulation square values are increased.

### GO Pathway enrichment analysis

To gain a deeper understanding of compartment change during aging, we firstly extracted genes that overlap with the genomic region of aging-related compartment. Then enrichment analysis were performed by “enrichGO” function of ClusterProfiler^62^ (v4.10.0) package (with parameters “ont = all”). Enriched terms with adjusted *p*-value < 0.01 were retained.

For TAD borders of interest, we performed GREAT^63^ analysis using rGREAT(v2.5.7) package (with default parameters). Enriched terms with HyperFDR < 0.01 were retained.

### Ensembl dscHi-C Hi-C data analysis

#### P(s) curve

The contact frequency vs. genomic distance decay curve was calculated by cooltools^64^ (https://github.com/open2c/cooltools, v0.5.4) ‘expected-cis’ function at 1-kb resolution.

#### A/B compartment

The A/B compartment (first eigenvalue of eigenvector decomposition on observed-over-expected contact matrix) values were calculated by cooltools ‘eigs-cis’ function at 100-kb resolution. Resolution was set to be 500 kb for metacells. Saddle plot was generated by saddle function at 500-kb resolution with parameters “qrange” setting to 0.02 to 0.98. Thus, each bin can be classified and compute saddle strength between each type of bin.

#### Insulation

The insulation scores were calculated by cooltools ‘insulation’ at either 10-kb or 25-kb resolution, the 10× window size insulation score was used to calculate pairwise correlation.

#### Chromatin loop identification and pile-up

The chromatin loops were detected using the cooltools ‘dots’ function with parameters ‘--fdr 0.01, diag_width = 10000000, tile_size = 5000000’ on bulk Hi-C data on mESC^65^. The pile-up analysis was performed by coolpuppy package (https://github.com/open2c/coolpuppy/tree/master, v0.9.7).

#### Contact map visualization

The contact maps were visualized by Juicebox^66^ (v1.11.08) or cooltools on a balanced contact matrix.

### Genomic track visualization

The ATAC-seq, H3K27ac ChIP-seq, aggregated dscHi-C multiome RNA-seq profiles, and bulk RNA-seq profiles were visualized by WashU epigenome browser (http://epigenomegateway.wustl.edu/browser/).

### Published datasets

mESC bulk Hi-C data were downloaded from 4DN database (“4DNFIC21MG3U_mESC_Oct4p_G0G1.mcool”), the GM12878 bulk Hi-C data were downloaded from 4DN database (“4DNFIXP4QG5B.mcool”). The mESC ATAC-seq and H3K37ac ChIP-seq data were downloaded from GEO under accession number GSE155089 (“GSM4694523_ATAC_ESC_rep1.bigWig”) and GSE155062 (“GSM4694560_H3K27ac_ESC.bigWig”), respectively. The mESC bulk RNA-seq data were downloaded from GEO under accession number GSE176044 (“GSE176044_mesc_bulk_rnaseq_gene_counts.csv.gz”).

## Supplementary information

Supplementary Material

Supplementary Table 1

Supplementary Table 2

## Data Availability

The raw sequencing data have been deposited in the Sequence Read Archive, and the processed data generated in this study have been uploaded to the Gene Expression Omnibus under accession code GSE285812.

## Code availability

The code used in this study was deposited to Github, dscHi-C tools (https://github.com/MaoxuWang/dscHiC_analysis). All plots were generated with matplotlib (v3.7.0) and ggplot2 (v3.3.3).
