# Supplementary material for: Droplet-based high-throughput 3D genome structure mapping of single cells with simultaneous transcriptomics
Source: Cell Discov. 2025 Jan 21;11:8. doi: 10.1038/s41421-025-00770-8 (PMC11751028; doi:10.1038/s41421-025-00770-8)
Supplement: Supplementary file 1 — Supplementary Material [file 41421_2025_770_MOESM1_ESM.pdf]

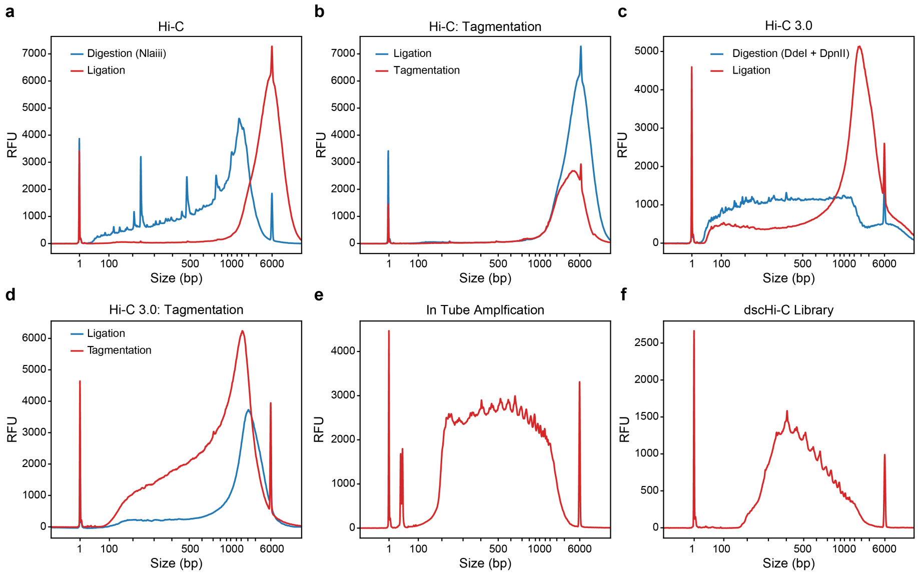

Fig. S1 | Demonstration of the feasibility of droplted based single-cell Hi-C. a, Fragment length distribution of chromatin after restriction enzyme digestion and after proximity ligation of Hi-C procedure on mESC. **b**, Fragment length distribution of chromatin after bulk tagmentaion on Hi-C ligated nuclei. **c**, The same as **a** for Hi-C 3.0 procedure on mESC. **d**, The same as **b** for Hi-C 3.0 ligated nuclei. **e**, Fragment length distribution of DNA after in tube amplification of bulk tagmented Hi-C 3.0 nuclei. **f**, Fragment length distribution of a representative dscHi-C library.

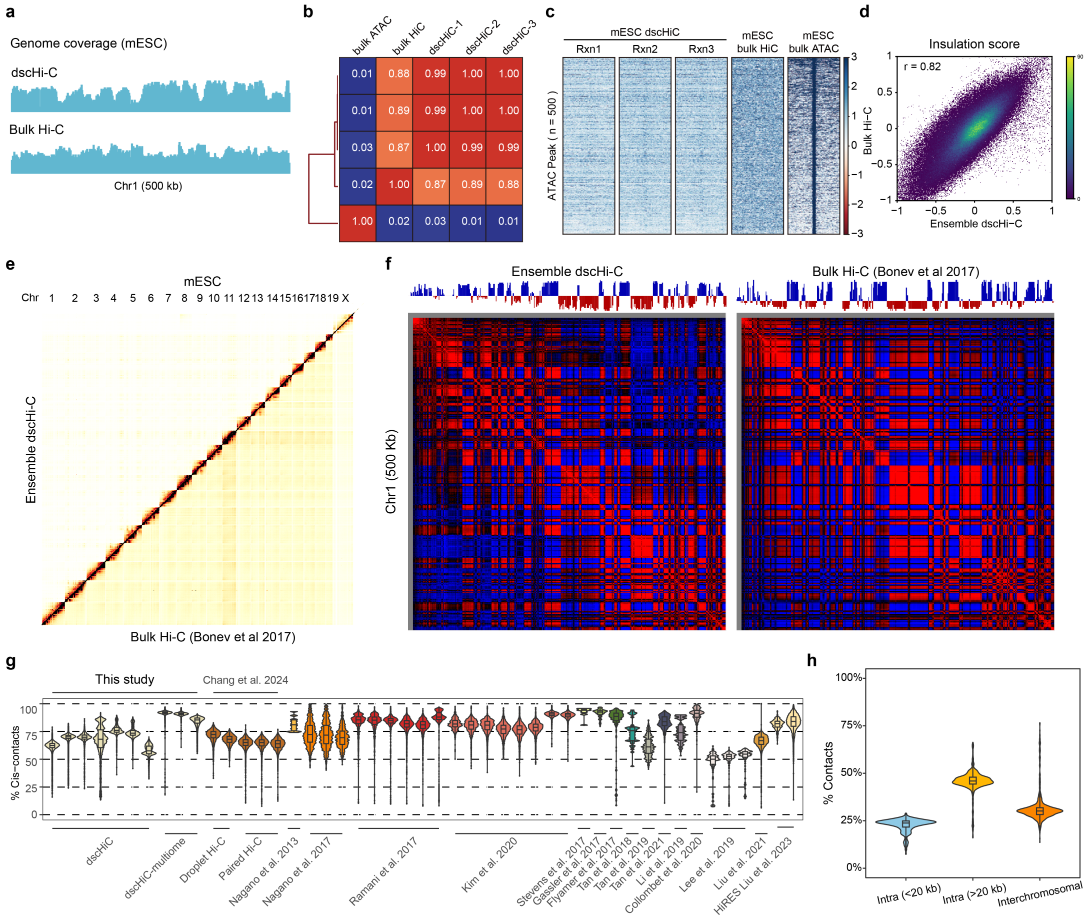

Fig. S2 | Validation of dscHi-C on mESC. a, Genome coverage tracks of ensemble dscHi-C and bulk Hi-C, chromosome 1 with 500 kb genomic bin. **b**, Pearson correlation heatmap between ensemble dscHi-C, bulk Hi-C and ATAC-seq of mESC, calculated at 500 bp genomic bin. **c**, Heatmap showing the enrichment around ATAC-seq peaks. **d**, Density plot showing the insulation scores of ensemble dscHi-C and bulk Hi-C. **e**, Contact maps of ensemble dscHi-C and bulk Hi-C, all chromosomes at 1 Mb resolution. **f**, Pearson correlation matrices of ensemble dscHi-C and bulk Hi-C, chromosome 1 at 500 kb resolution. Eigen value tracks shown at the top. **g**, Violin plot comparing the percentage of intrachromosomal contacts between dscHi-C and other published scHi-C datasets. **h**, Violin plot showing the composition of contacts of mESC.

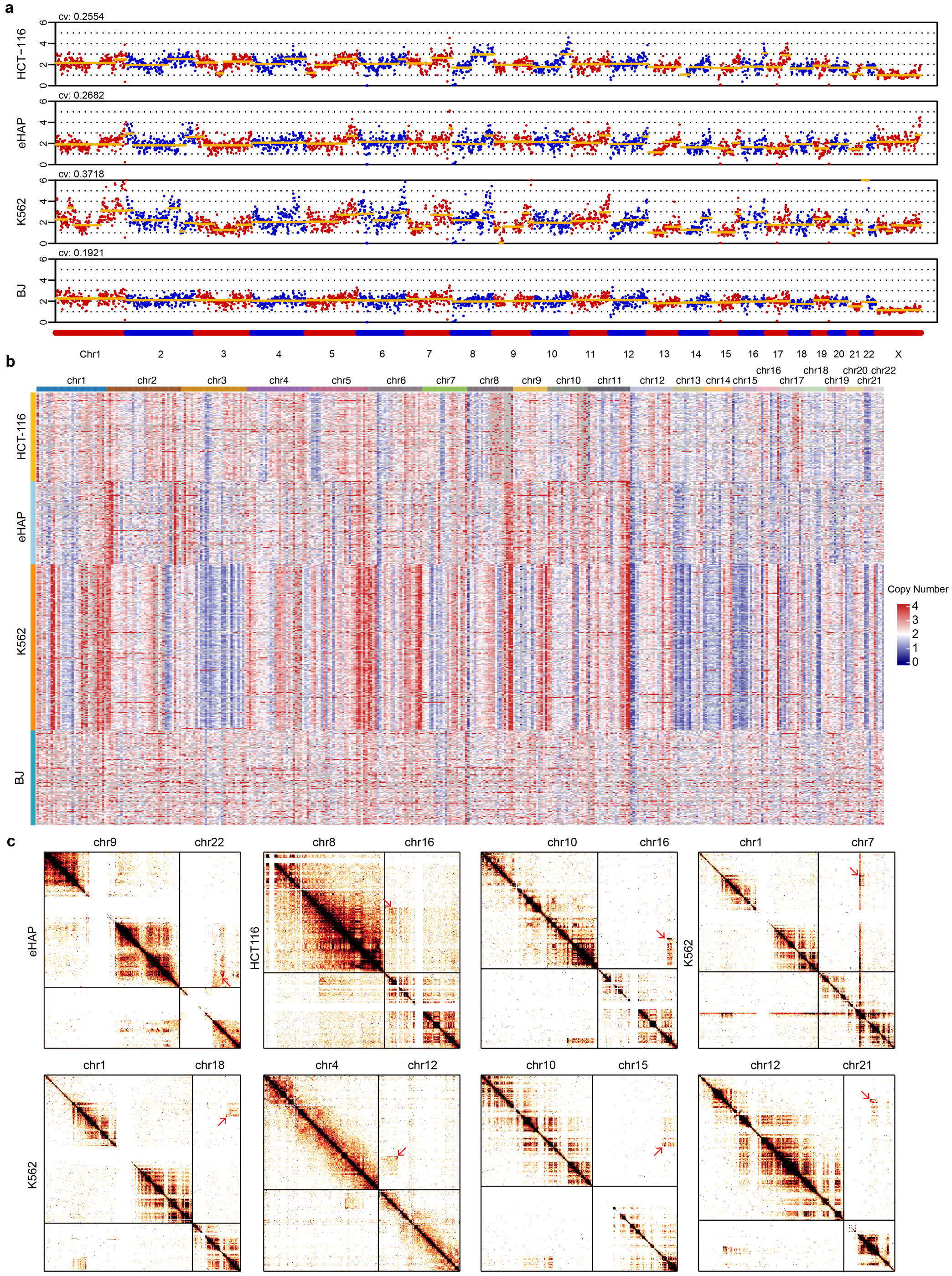

Fig. S3 |. dscHi-C precisely delineates copy number variation across multiple cell lines. a, Copy number of ensemble dscHi-C profiles of HCT-116, eHAP, K562 and BJ at 1 Mb genomic bin size. Only autosomes are shown. **b**, Heatmap showing the CNV of dscHi-C single-cell profiles of HCT-116, eHAP, K562 and BJ at 1 Mb genomic bin size. **c**, Contact maps showing that dscHi-C captured the known translocation events in the corresponding cancer cell lines.

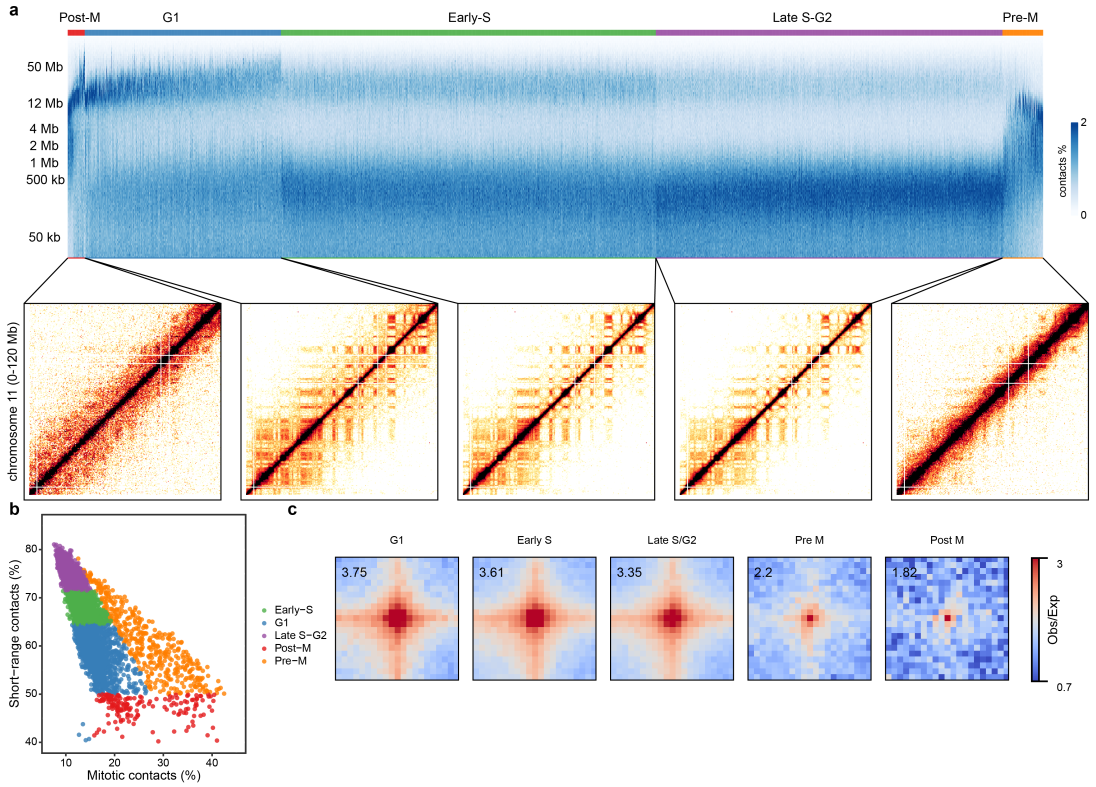

Fig. S4 | mESC dscHi-C data delineates cell cycle dependent chromatin structure reorganization. a, Single-cell dscHi-C profiles ordered *in silico* to infer cell cycle phasing (n = 11,000), the cell cycle was indicated at the top, ensemble contact maps of each cell cycle were shown at below. **b**, Scatter plot showing the percentage of short-range (< 2 Mb) and mitotic band (2–12 Mb) contacts of single-cell profiles. **c**, Aggregation of chromatin loops for each cell cycle at 10 kb resolution with 100 kb flanking, chromatin loop sets was detected on bulk Hi-C data.

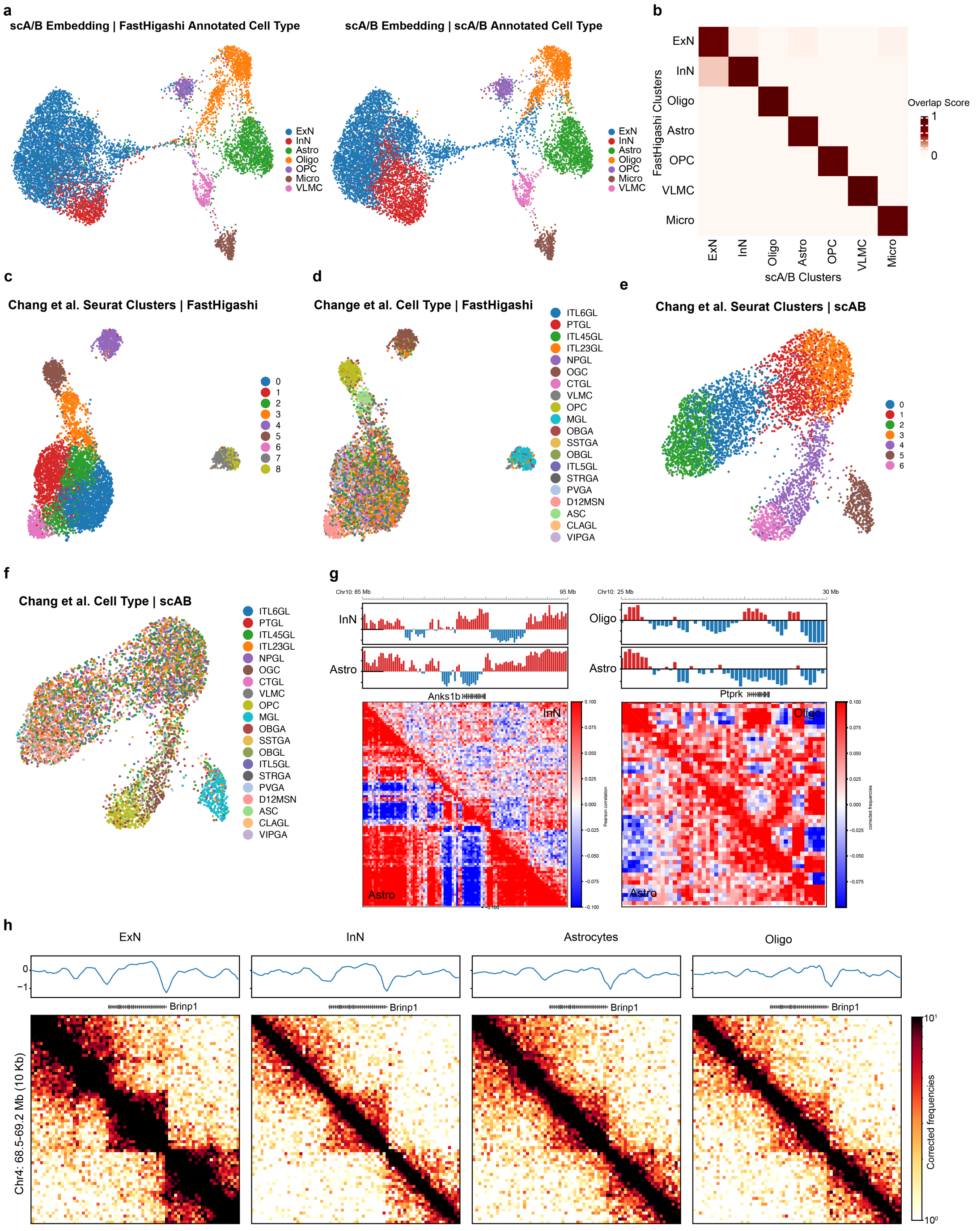

Fig. S5 | dscHi-C resolves major cell types in mouse brain cortex. a, Embedding of mouse brain dscHi-C data, embedding was performed by scA/B values defined by Dip-C. **b**, Confusion matrix of cell clusters in scA/B value embedding and FastHigashi embedding. **c**, UMAP embedding of Chang et al droplet Hi-C data from the mouse brain by Higashi, color by Seurat unsupervised clustering. **d**, The same as **c**, but the cell type annotation from Change et al was projected. **e**, UMAP embedding of Chang et al droplet Hi-C data from the mouse brain by scA/B value, color by Seurat unsupervised clustering. **f**, The same as **e**, but the cell type annotation from Change et al was projected. **g**, Pearson correlation matrix at two representative marker gene loci. **h**, Contact maps at Brinp1 gene locus, insulation score was plotted on the top.

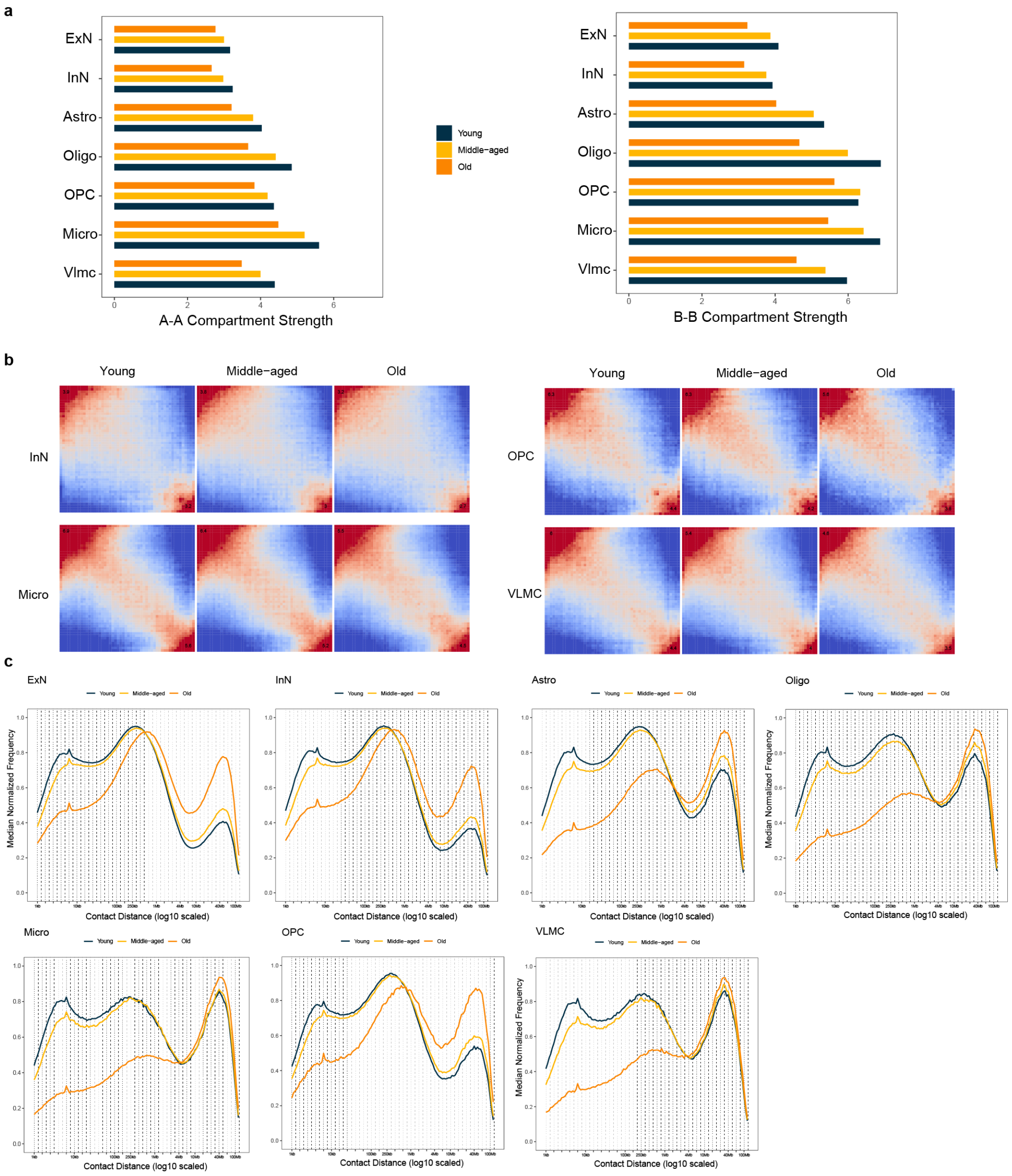

Fig. S6 | Compartmentalization and global chromatin interaction frequency changes during aging process. a, Barplot showing the quantitative compartmentalization weakening during aging. **b**, Saddle plot showing the weakening of compartmentalization. **c**, Contact frequency versus contact distance, showing the prominent changes in the global contact pattern.

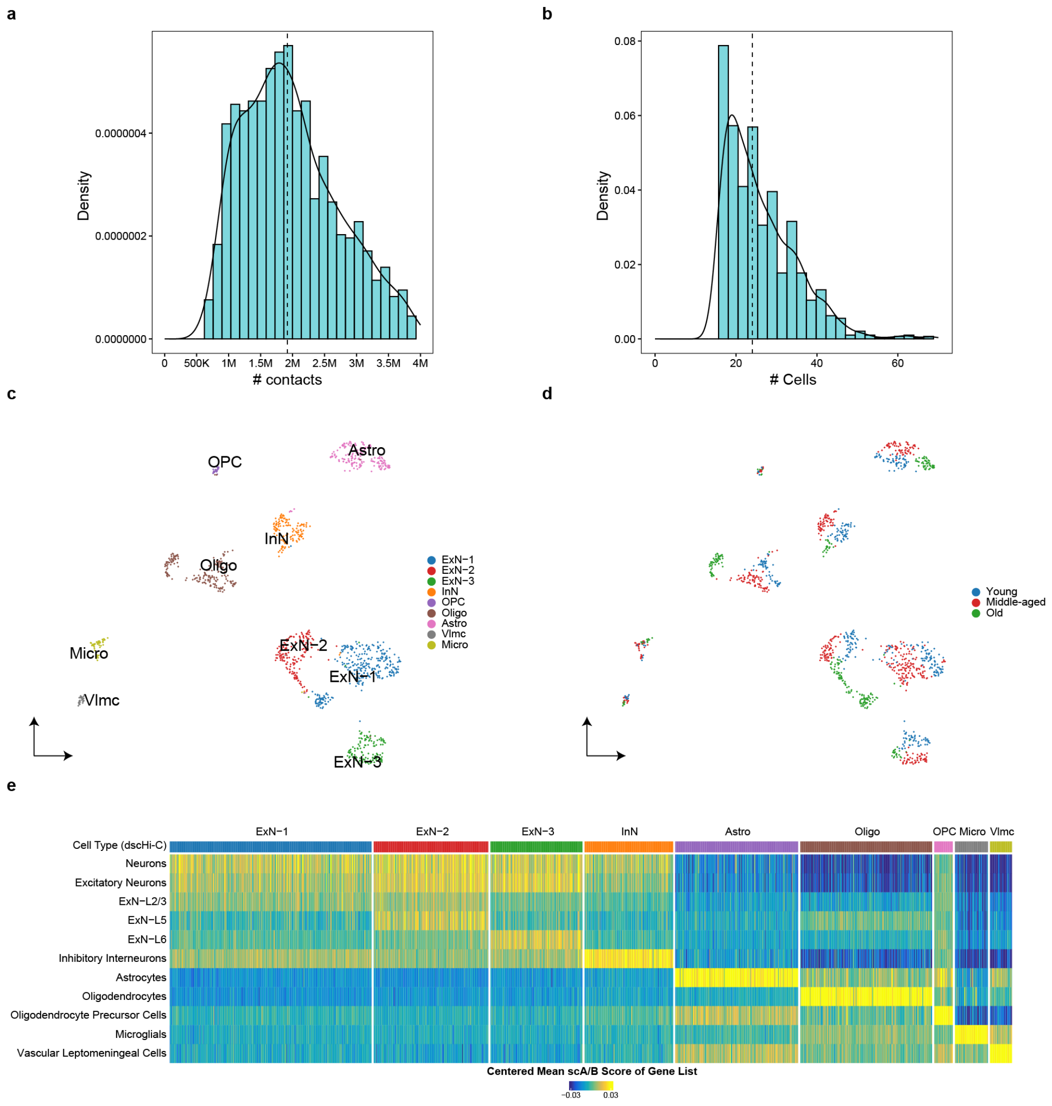

Fig. S7 | Metacell analysis of mouse brain aging dscHi-C data. a, Histogram showing the distribution of number of contact of metacells. **b**, Histogram demonstrates the distribution of number of cells merged for metacell. **c-d**, UMAP embedding of metacells from mouse brain, colored by cell types (**c**) and colored by age (**d**). **e**, Heatmap showing the scA/B values of marker genes of metacells.

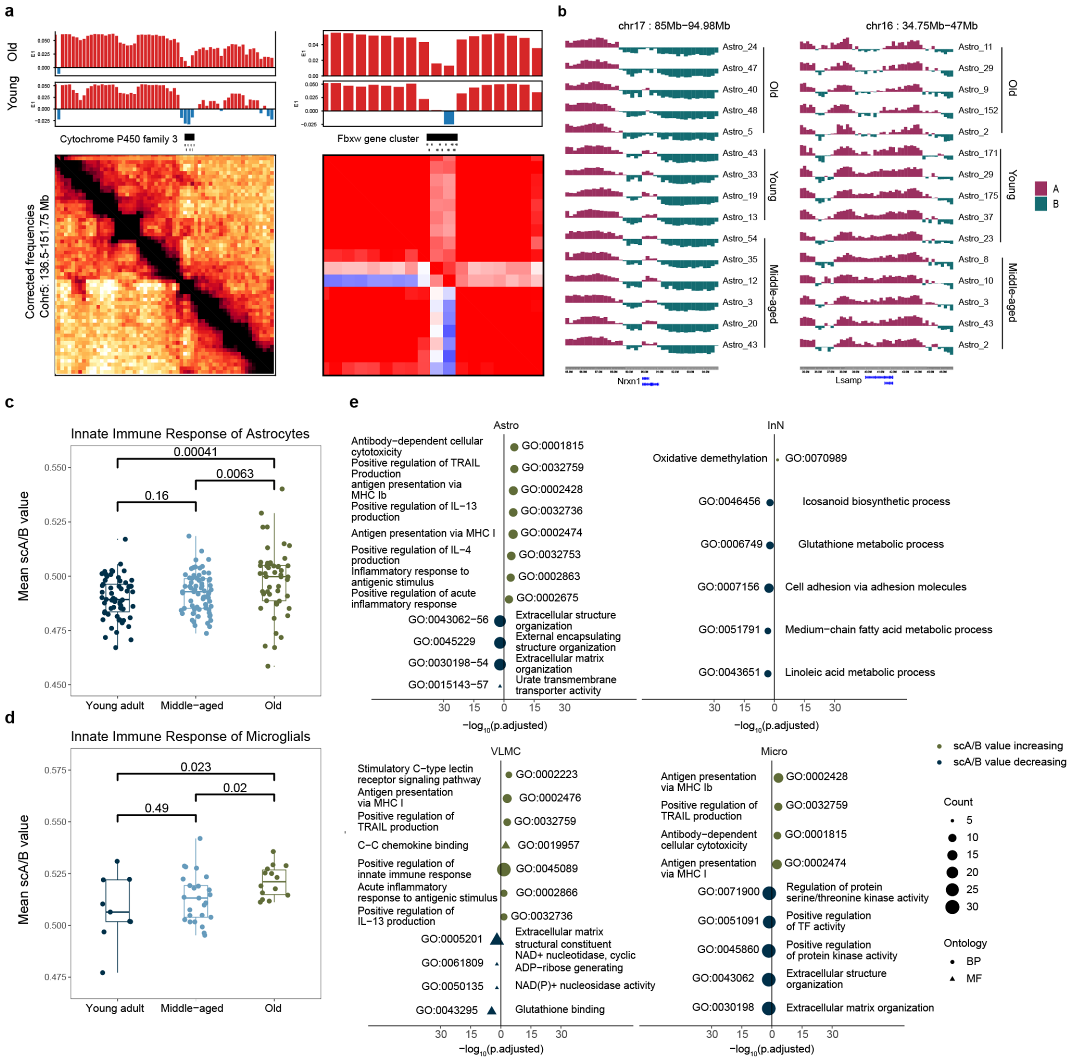

Fig. S8 | Aging-related compartment changes. a, Examples of gene clusters transitioning from B to A compartments in oligodendrocytes during aging. **b**, Representative genes in astrocytes that exhibit compartmental shifts during aging. **c-d**, Boxplots demonstrating the increase in scA/B values associated with the innate immune response pathway in astrocytes (**c**) and microglia (**d**). **e**, Analysis of enriched molecular functions and pathways associated with genomic bins that display compartment changes.

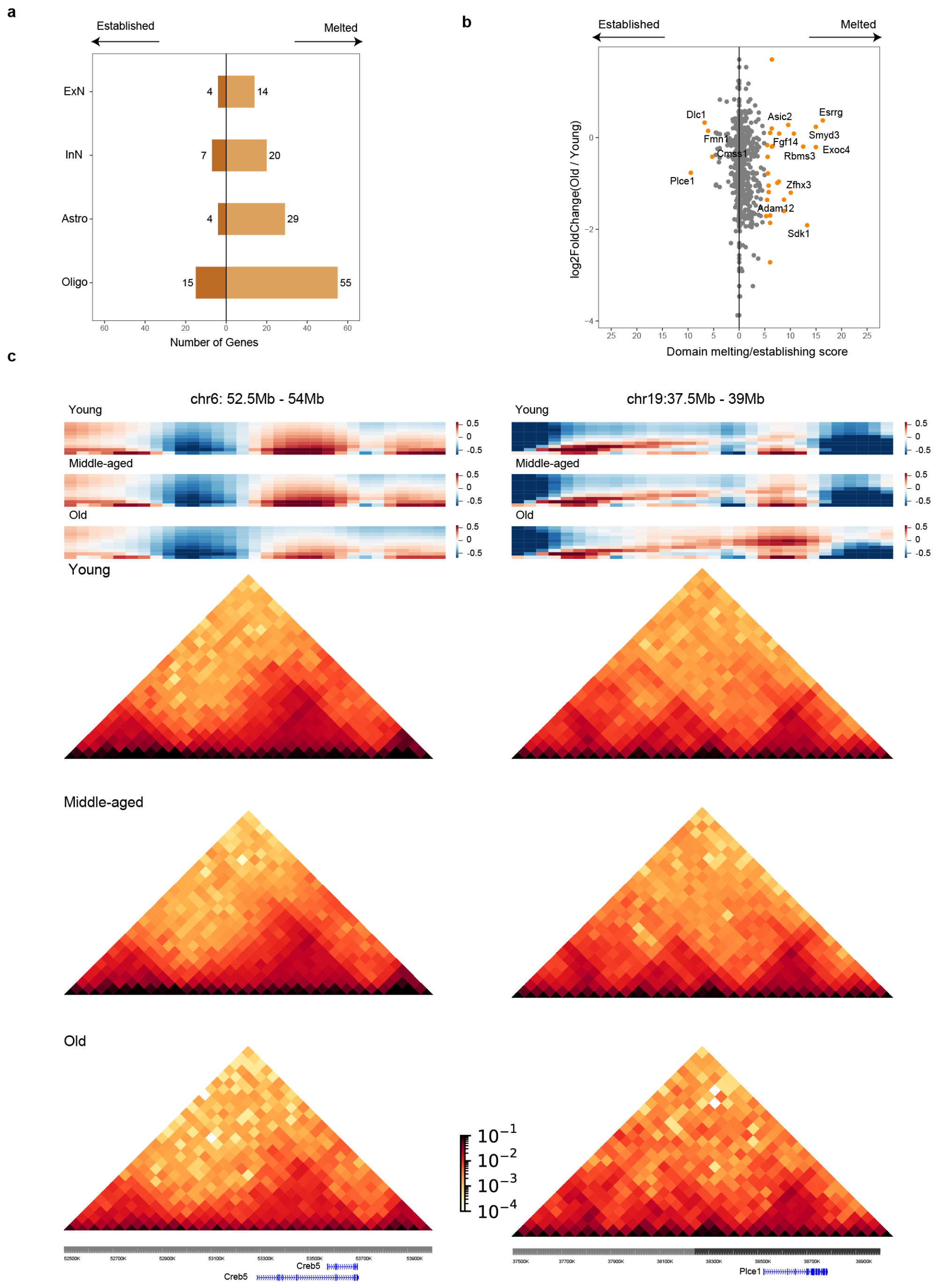

Fig. S9 | Long genes that show prominent insulation changes during physiological aging process. a, Bar plot summarize the number of genes show melting of establishment in each cell types during aging. **b**, Scatter plot showing the genes that show significant insulation changes along with the expression changes. **c**, Representative genes show melting (left) and establishment (right) in astrocytes during aging.

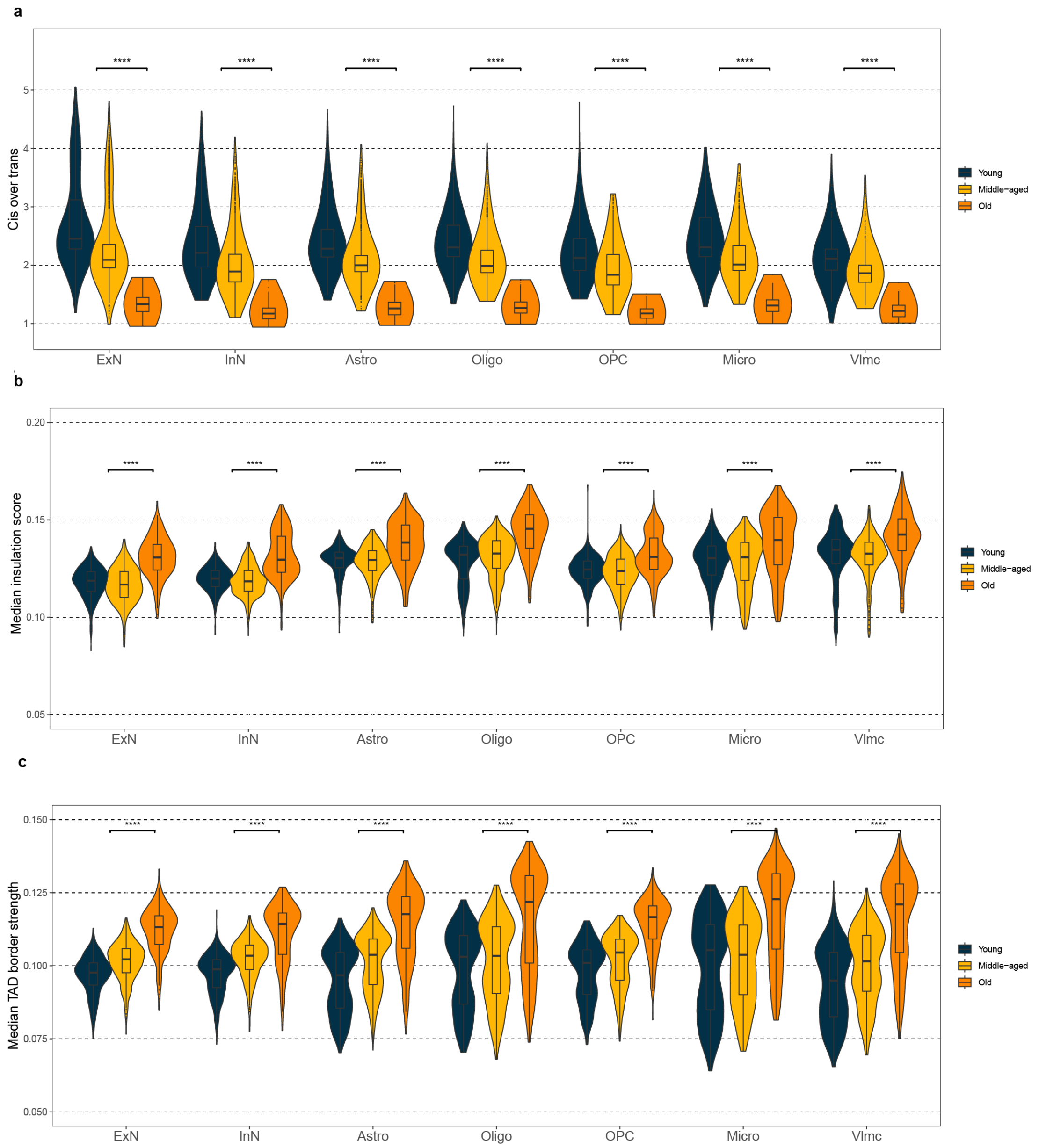

Fig. S10 | The increase of insulation and inter-chromosomal interactions during aging. a, Violin plots illustrating the increase in chromosomal intermingling with age. **b-c**, Violin plots depicting the insulation scores of topologically associating domain (TAD) boundaries (**b**) and TAD strength (**c**) across different ages.

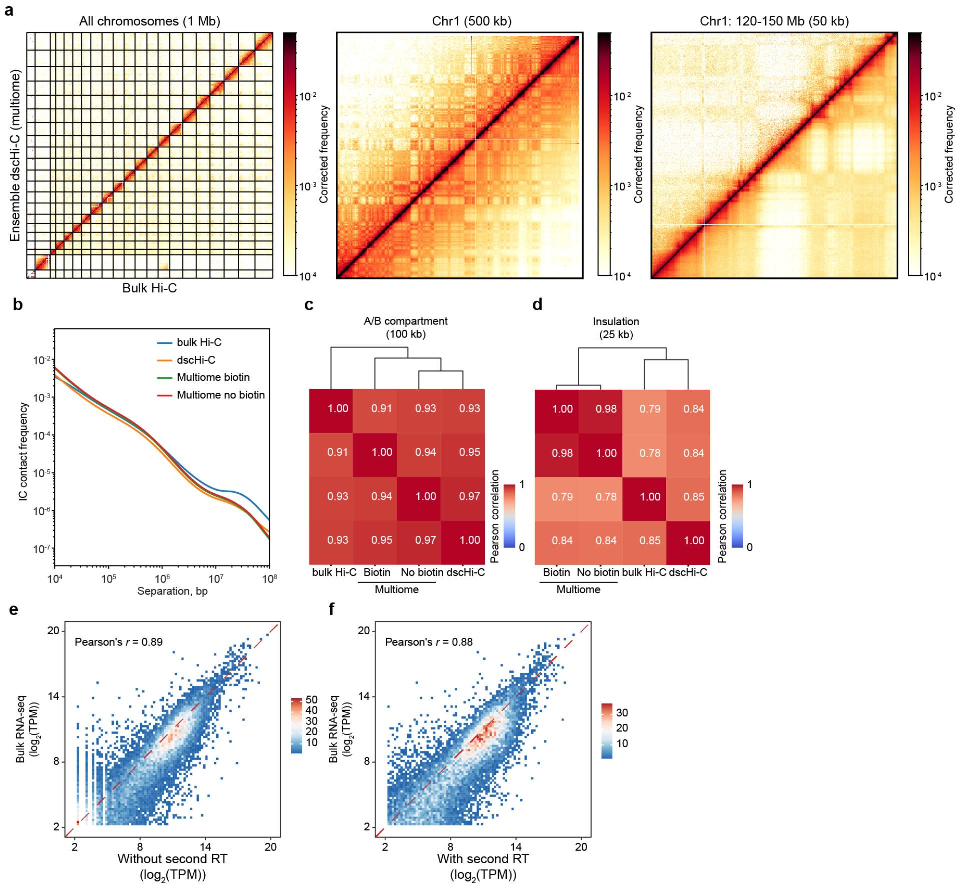

Fig. S11 | Validation of dscHi-C Multiome procedure in mESC. a, Contact maps comparing dscHi-C multiome and bulk Hi-C data. From left to right: all chromosomes at 1 Mb resolution, chromosome 1 at 500 kb resolution, and the region chr1: 120–150 Mb at 50 kb resolution. **b**, Contact frequency versus genomic distance decay curves. **c**, Heatmap illustrating the pairwise Pearson correlation of eigenvalues among dscHi-C, dscHi-C multiome (with and without biotin enrichment), and bulk Hi-C datasets at 100 kb resolution. **d**, Heatmap presenting the pairwise Pearson correlation of insulation scores among dscHi-C, dscHi-C multiome (with and without biotin enrichment), and bulk Hi-C datasets at 25 kb resolution. **e-f**, Density plot showing the correlation between aggregated single-cell RNA-seq profiles without second reverse transcription (**e**) or single-cell RNA-seq profiles with second reverse transcription (**f**) and bulk RNA-seq.

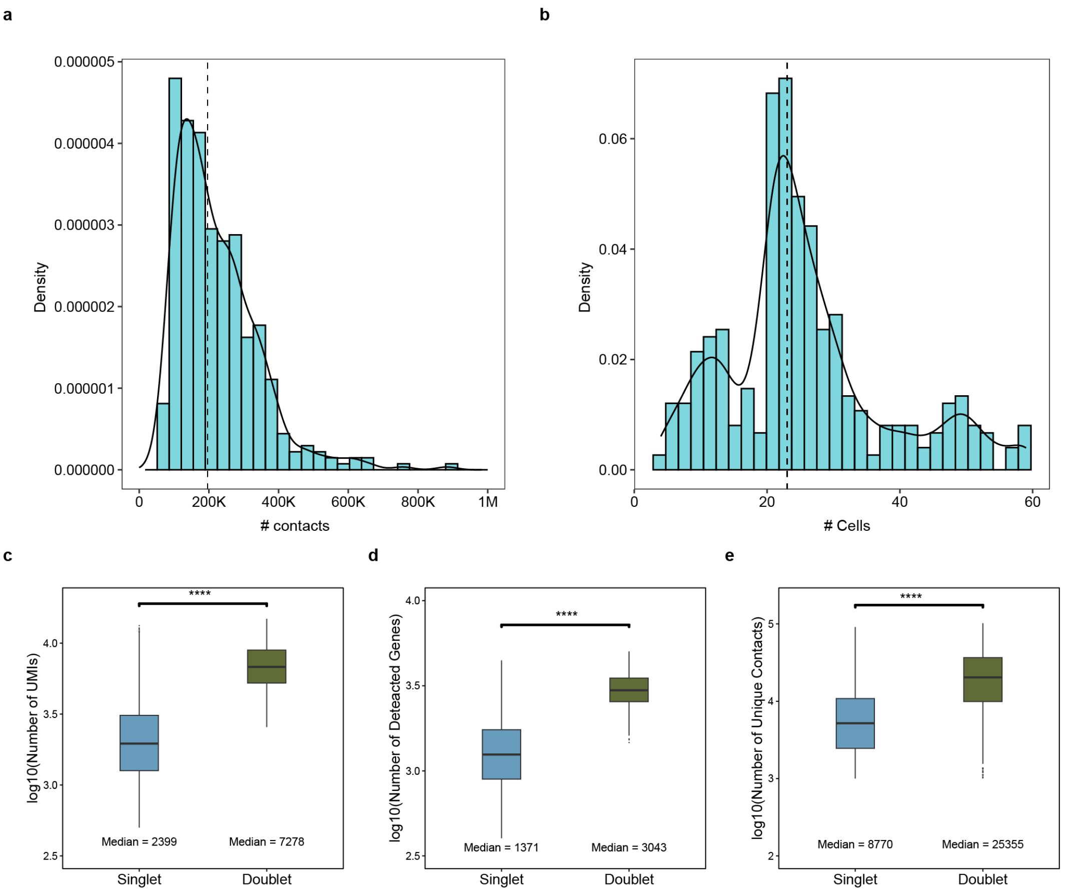

Fig. S12 | Doublets removal in dscHi-C Multiome data. a, Histogram illustrating the distribution of contact number among metacells in the dscHi-C-Multiome dataset from the adult mouse brain. **b**, Histogram depicting the distribution of the number of cells comprising each metacell. **c-e**, Boxplots comparing the number of Unique Molecular Identifiers (UMIs) (**c**), the number of detected genes (**d**), and the number of unique contacts (**e**) between singlets and doublets.
